# Captopril alleviates epilepsy and cognitive impairment by attenuation of C3-mediated inflammation and synaptic phagocytosis

**DOI:** 10.1186/s12974-022-02587-8

**Published:** 2022-09-14

**Authors:** Xinyan Dong, Jianchen Fan, Donghui Lin, Xuehui Wang, Haoyu Kuang, Lifen Gong, Chen Chen, Jie Jiang, Ningxiao Xia, Dahong He, Weida Shen, Peifang Jiang, Rong Kuang, Linghui Zeng, Yicheng Xie

**Affiliations:** 1 Department of Neurology, The Children’s Hospital, Zhejiang University School of Medicine, National Clinical Research Center for Child Health, Hangzhou, 310052 China; 2 School of Medicine, Zhejiang University City College, Hangzhou, 310015 China; 3 School of Life Science, Lanzhou University, Lanzhou, 730000 China; 4 Zhejiang Institute for Food and Drug Control, Hangzhou, 310052 China

**Keywords:** Epilepsy, Cognitive deficits, Captopril, Complement 3, Glial activation, Synaptic phagocytosis

## Abstract

Evidence from experimental and clinical studies implicates immuno-inflammatory responses as playing an important role in epilepsy-induced brain injury. Captopril, an angiotensin-converting enzyme inhibitor (ACEi), has previously been shown to suppress immuno-inflammatory responses in a variety of neurological diseases. However, the therapeutic potential of captopril on epilepsy remains unclear. In the present study, Sprague Dawley (SD) rats were intraperitoneally subjected to kainic acid (KA) to establish a status epilepticus. Captopril (50 mg/kg, i.p.) was administered daily following the KA administration from day 3 to 49. We found that captopril efficiently suppressed the KA-induced epilepsy, as measured by electroencephalography. Moreover, captopril ameliorated the epilepsy-induced cognitive deficits, with improved performance in the Morris water maze, Y-maze and novel objective test. RNA sequencing (RNA-seq) analysis indicated that captopril reversed a wide range of epilepsy-related biological processes, particularly the glial activation, complement system-mediated phagocytosis and the production of inflammatory factors. Interestingly, captopril suppressed the epilepsy-induced activation and abnormal contact between astrocytes and microglia. Immunohistochemical experiments demonstrated that captopril attenuated microglia-dependent synaptic remodeling presumably through C3–C3ar-mediated phagocytosis in the hippocampus. Finally, the above effects of captopril were partially blocked by an intranasal application of recombinant C3a (1.3 μg/kg/day). Our findings demonstrated that captopril reduced the occurrence of epilepsy and cognitive impairment by attenuation of inflammation and C3-mediated synaptic phagocytosis. This approach can easily be adapted to long-term efficacy and safety in clinical practice.

**Graphical Abstract:**

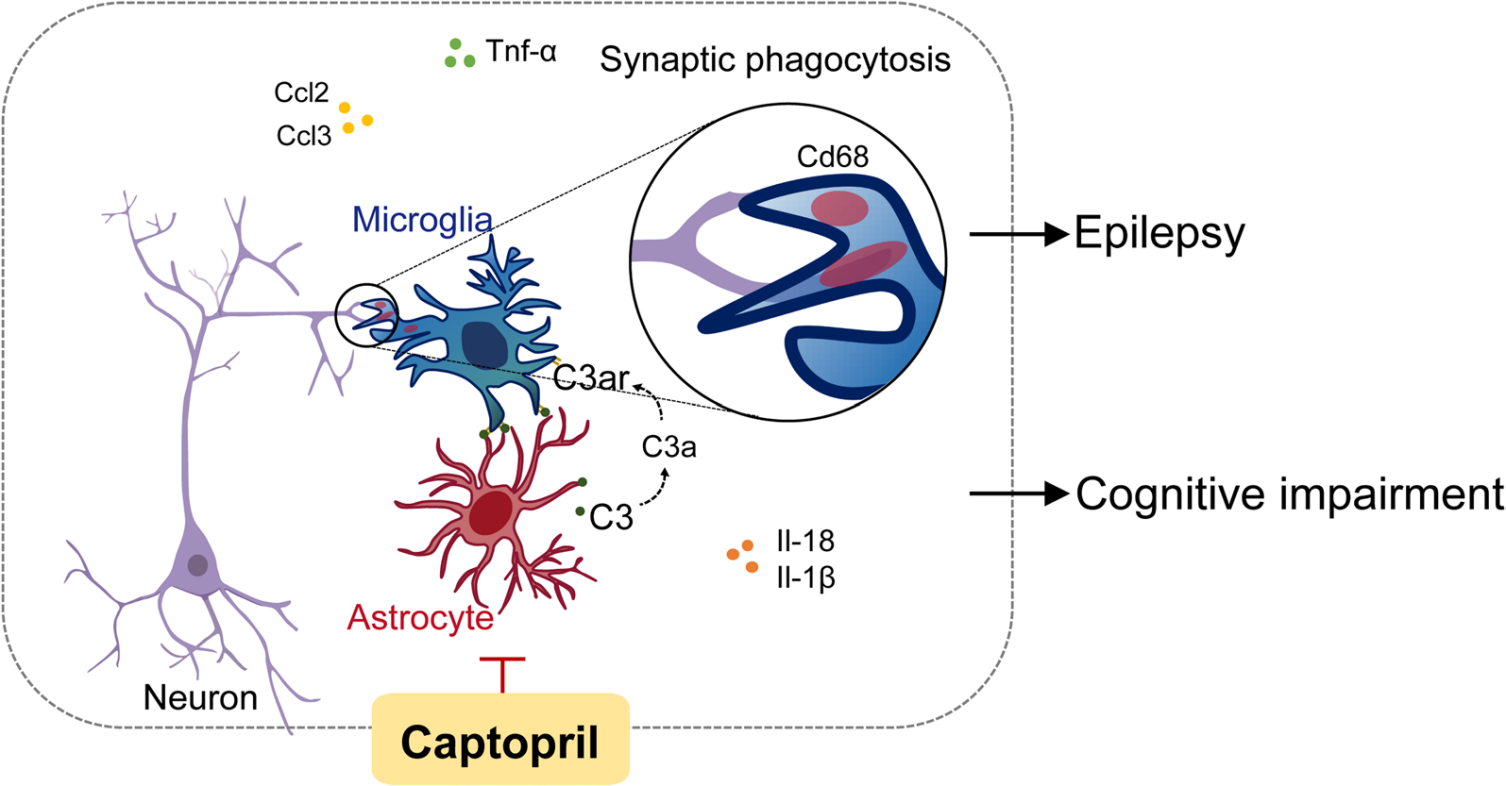

## Introduction

Epilepsy is a brain dysfunction disease characterized by sudden abnormal high-frequency discharges in local brain lesions and spread to surrounding tissues [1]{Cihan, 2018 #7}, which may be accompanied by transient movement, sensory, conscious, and autonomic nerve dysfunction. Temporal lobe epilepsy (TLE), which originates in the hippocampus or amygdala, is the most prevalent form of seizure, accounting for 60% of the overall incidence of epilepsy [2]. If not treated immediately, severe neurological damage associated with epilepsy increases, including the development of cognitive comorbidities, such as hippocampal-dependent spatial learning and memory deficits [3]. Current therapeutics (such as carbamazepine, sodium channel blockers, etc.) for epilepsy aim to inhibit the abnormal discharge of neurons to control the initiation and progression of seizures. Unfortunately, about one-third of patients still fail to achieve control and evolve into drug-resistant epilepsy, manifested by multiple seizures types and progressive cognitive and developmental deterioration [4]. Therefore, it is essential to find new therapeutic targets for epilepsy and its related comorbidities [5].

It is becoming clear that brain inflammation promotes neuronal hyperexcitability and seizures. Dysregulation in the glia immune-inflammatory function is a common factor in the human brain and experimental epilepsy models [6]. Onset of epileptogenesis is strongly interconnected with the production of proinflammatory cytokines in microglia and astrocytes, including interleukin-1β (Il-1β), Il-6, tumor necrosis factor-α (Tnf-α) and other cytotoxic factors, which initiate further inflammatory cascades and contribute to impairment of neuronal function [7–9]. Moreover, microglia and astrocytes undergo structural remodeling towards proinflammatory phenotypes under the stimulation of inflammatory factors [10] and dysfunctional of energy homeostasis in astrocytes can contribute to neuronal hyperexcitability in the pathophysiological processes that lead to the development of epilepsy [11]. Chronically activated astrocytes and microglia are prominent features of epileptic foci that can irreversibly damage the brain, contributing to the pathophysiology of epilepsy and cognitive impairment [12, 13]. Consequently, targeting molecules with activation of glial cells and production of inflammatory factors is currently in development as promising antiepileptogenic therapeutic approach.

Accumulated evidence shows that the complement system plays a critical role in glia-mediated effects during physiological and pathological processes of neuro-immune diseases [14, 15]. Recent studies also prove that RNA transcripts and protein levels of C1q and C3 are increased in the hippocampus of patients and animal models of epilepsy [16]. The complement components C1q and C3 mediate synapse elimination during development and neurodegeneration through C3-dependent microglia phagocytosis [16, 17]. In this signaling pathway, complement protein C3 splits into C3a and C3b. C3a covalently binds to the specific receptor C3ar located on the surface of macrophages and microglia, exerting a series of inflammatory reactions including synaptic phagocytosis and the release of proinflammatory factors and chemokines, which can directly cause neuronal damage and cell death in the diseases [18, 19].

Although the relationship between epilepsy and inflammation has been well established, attempts to prevent epileptogenesis by modulating various inflammation and immune-related abnormalities have had only limited success. Research shows that the renin–angiotensin system (RAS) system participates in producing inflammatory cytokines both in the peripheral and central nervous system (CNS) [20]. Angiotensin-converting enzyme (ACE) converts angiotensin I (Ang I) to Ang II, which stimulates angiotensin type 1 receptor (AT_1_R) on the surface of astrocytes and microglia resulting in enhanced production and release of multiple proinflammatory factors [21]. It has been established that Ang II levels are elevated in epilepsy, causing dysregulation of the neuronal–microglial signaling and production of proinflammatory factors Tnf-α [22]. Ang II also stimulates several cellular inflammatory responses, including increased expression of vascular cell adhesion molecule 1 (Vcam-1), Il-1β, CC chemokine ligand (Ccl) 2 and Ccl5, thus leading to an accumulation of immune cells [23]. Treatment of angiotensin-converting enzyme inhibitors (ACEi) can prevent proconvulsant activation exerted by Ang II, thus reducing the detrimental consequences resulting from epilepsy [24]. Captopril is one of the ACEis that reduces the formation of Ang II and exerts beneficial anti-inflammatory effects in lipopolysaccharide (LPS)-treated glial culture [25]. Continuous blocking AT_1_R was effective against neuronal loss and exerted neuronal protection in the hippocampus in rats with epilepsy [26, 27]. However, the specific effects and molecular mechanisms of ACEis on immuno-inflammatory responses during epilepsy remain obscure [28, 29]. Interestingly, the latest research has demonstrated that ACE can directly cleave the downstream product C3f of C3 through two homologous catalytic activity domains (N- and C-domains). The cleavage of C3f inversely promotes the synthesis of complement C3, which was significantly suppressed in ACE knockout mice [30]. In addition, ACE inhibition by systemic administration of captopril ameliorated cognitive function and neurodegeneration in several Alzheimer’s disease (AD) models by reducing the expression of complement protein C3 [31]. Therefore, studying the effects of ACE inhibitors on the C3-dependent glial activation is of great significance for the clinical medication for epilepsy.

In the present study, we identified captopril as a potential complement signaling blocker and tested its efficacy in preventing epilepsy and cognitive impairment. We found that the captopril treatment significantly protected rats from long-term complications of epilepsy. Mechanistically, continuous captopril therapy inhibited glial activation and aberrant synaptic phagocytosis in the hippocampus by reducing complement C3–C3ar expression in epilepsy. Furthermore, when C3a, an active cleavage product of C3, was administered intranasally, we found the beneficial effects of captopril on the prevention of epilepsy and its related complications were partially blocked. This result indicated a causal relationship wherein the therapeutic effects of captopril treatment were mediated by suppressing the C3–C3ar signaling pathway. Taken together, these data suggest that hyperactivation of C3–C3ar is critical for the detrimental effects associated with synaptic phagocytosis in epilepsy. Thus presenting pharmacological inhibition of C3–C3ar through ACEi is a promising target for the treatment of epilepsy and cognitive complications.

## Materials and methods

### Animals and seizure monitoring

Animal protocol was in accord with the guidelines of Zhejiang University Animal Care. Male Sprague Dawley (SD) rats (120–140 g) were purchased from Shanghai SLAC Laboratory Animal Co., Ltd (certificate: SCXK 2017-0005) and were raised at room temperature of 22 ± 1 °C with food and water ad libitum.

Rats were randomly divided into 3 groups: control group (with saline intraperitoneal (ip) injection), KA group (with 14 mg/kg kainite acid (ab120100, Abcam, USA) ip injection), and KA + Cap group (with 50 mg/kg captopril ip injection). Seizure severity was rated by the Racine scale: category 1, immobility and facial twitch; category 2, head nodding; category 3, forelimb clonus; category 4, rearing; and category 5, rearing and falling. The onset of seizures was defined as the beginning of category 4–5 seizures. Seizures were terminated by 0.1% diazepam (10 mg/kg) after 3 h. If the animals did not develop category 4–5 seizures, every 1 h after a KA injection, an additional injection at 50% of the initial dose was given until they developed stage 4–5 seizures. If the animals did not develop stage 4 seizures after 2 additional applications of KA, they were excluded from the subsequent study.

The model of epilepsy has been described extensively [32]. Epilepsy develops after nonconvulsive status epilepticus lasting for at least 3 h, initially induced by 14 mg/kg KA injection intraperitoneally. Recurrent seizures usually start 1 week after the induction of status epilepticus and can last for at least up to 2 months. The baseline of epilepsy in each rat is stable and reproducible, and seizures do not occur in clusters, as assessed by measuring the frequency and duration of EEG ictal events from week 2 to 7 after the development of status epilepticus. This feature is a key prerequisite for reliable pharmacological studies.

### Systemic captopril administration

Captopril (C4042, Sigma, USA) was freshly prepared in 0.9% NaCl. The KA-injected rats received daily doses of vehicle (0.9% NaCl) or captopril (50 mg/kg by ip injection from the 3rd day after the KA injection) for 7 weeks. Rats were weighed weekly. The selection of captopril dosing was based on previous studies [33–35].

### Intranasal treatment of C3a

Purified human C3a peptide (A113, Complement Technologies, USA) was diluted in sterile phosphate-buffered saline (PBS) to a concentration of 200 nM, and a total of 20 μL (10 μL/nostril; corresponding to 1.13 mg/kg body weight) of PBS was given intranasally (in) to awake, hand-restrained rat held in a supine position. Solutions were administered through a pipette tip, drop-wise into 5-μL portions at a time interval of 1 min to allow for absorption [36]. C3a or PBS was given daily from Day 3 to 49 after the KA induction and along with the captopril treatment. Rats were assigned to C3a or PBS treatment using randomization stratified by body weight to avoid potential confounding effects of body weight on behavioral performance. The investigation carrying out behavioral studies and data analysis were blinded to the treatment group.

### Total RNA isolation and quantitative polymerase chain reaction

Total RNA was extracted from the samples using TRIzol (10296010, Invitrogen, USA), according to the manufacturer’s instructions. The total RNA was quantified using a spectrophotometer (Nanodrop 2000, Thermo Fisher, USA). All the samples presented 260/280 nm ratios between 1.8 and 2.0.

RNA samples of the hippocampus from 3 rats from each group were collected and sent to WuHan Bioacme Biological Technologies Corporation (Wuhan, China). After the initial Quality Control (QC) assessment (RNA concentration and integrity were assessed by the Nanodrop spectrophotometer, the samples presented 260/280 nm ratios between 1.8 and 2.0. Later, RNA integrity was assessed by an Agilent Bioanalyzer Nano-ChIP, relative integrity index (RIN) of RNA samples were ≥ 9). The mRNA was then enriched by oligo (dT) beads. After concentrations were determined with an adaptor-specific q-PCR kit, equimolar samples were pooled and clustered for sequencing on the HiSeq2000 (Illumina, USA).

The fragments per kilobase of transcript per million mapped reads were used to estimate gene expression levels in each sample. Differential gene analysis was carried out using the DESeq2 package in the R environment [37]. Differences with *p* value < 0.05 and log2 |fold change|> 1 were considered significant. Gene Ontology (GO) and Kyoto Encyclopedia of Genes and Genomes (KEGG) pathway analysis were performed using DAVID (https://david.ncifcrf.gov/) on the rat genome. The gene set enrichment analysis (GSEA) was performed using the ranked list of genes based on the log-fold change.

### Primer design and real-time quantitative PCR (RT-qPCR)

Gene sequences were obtained from the Ensemble Genome Browser database and primer pairs were designed aligned in different exons using Primer 3 (Table. 1). Quality and specificity were evaluated using Primer-BLAST, respectively. Primers were synthesized by IDT—Integrated DNA Technologies (Sangon Biotech).

**Table 1:** Sequences of the primers

Gene	Primer	Sequence
Il-1β	Forward	TGACTCGTGGGATGATGACG
	Reverse	AGGCCACAGGGATTTTGTCG
Tnf-α	Forward	ATGGGCTCCCTCTCATCAGT
	Reverse	CAAGGGCTCTTGATGGCAGA
Il-18	Forward	GAGCTGGAGGACAAGGGAAC
	Reverse	GCCCGTTATGGTGGACAGAA
Ccl2	Forward	GCCTGTTGTTCACAGTTGCT
	Reverse	ACCCATTCCTTATTGGGGTCAG
Ccl3	Forward	TGCTGCTTCTCCTATGGACG
	Reverse	AGATCTGCCGGTTTCTCTTGG
Cxcl13	Forward	CAGTGGCAGGGATTCACTTCA
	Reverse	ACTTGGGGACACCCTTTAGC
C3	Forward	TGGGCAAGACAGTCGTCATC
	Reverse	TCCATCAGCACTTTTCGGCT
C3ar	Forward	ACTGGCCCTATGGCTTGTTC
	Reverse	TCATCCGGGAAATCATGGGC
Iba1	Forward	GCCAGAGCAAGGATTTGCAG
	Reverse	CGTCTTGAAGGCCTCCAGTT
Gfap	Forward	TGGCCACCAGTAACATGCAA
	Reverse	GAGTGCCTCCTGGTAACTCG
β-actin	Forward	ATATCGCTGCGCTCGTCGT
	Reverse	GAGGCATACAGGGACAACACA

Reverse transcription reaction was carried out with total RNA from all samples using the PrimeScript™ RT reagent Kit (RR037A, Takara Bio, Japan) according to the manufacturer’s instruction. RT-qPCR was performed using the LightCycler® 480 Instrument II (Roche, Switzerland) and the protocol of the TB Green® Premix Ex Taq™ (RR420A, Takara Bio, Japan), and the cycling conditions were as follows: an initial 30 s denaturation at 95 °C and 35 cycles (5 s at 95 °C and 40 s at 60 °C). The efficiency of the primers was verified through a dilution series curve. The housekeeping gene β-actin was selected as a reference gene. Transcript levels were normalized using the control group as the calibrator. All samples were run into triplicates, and the results were analyzed using the 2^(−ΔΔCt)^ method.

### Immunostaining and image analysis

After perfusion with 4% paraformaldehyde (PFA), the rat brains were fixed with 4% PFA overnight at 4 °C and then transferred into 30% sucrose solution. Brain sections (30 μm) were cut using a sliding microtome (HM525, Microm, Germany) and stored at − 20 °C in a cryoprotectant. For each experiment, 3–4 corresponding sections between 4.56 and 3.60 mm posterior from the bregma in one hemisphere of the rat brain were collected from 3 animals per group. After washing in PBS, sections were blocked with QuickBlock™ blocking buffer for immunol staining (P0260, Beyotime, China) for 1 h and then were incubated in primary antibody diluted in the blocking solution overnight at 4 °C (mouse anti-Cd68, 1:100, ab955, Abcam, UK; rabbit anti-Iba1, 1:1000, PTR2404, Wako, Japan; chicken anti-Gfap, 1:1000, ab5541, Abcam, UK; mouse anti-C3, 1:1000, sc-28294, Santa Cruz, USA; mouse anti-C3ar, 1:100, HM3028, Hycult, USA; guinea pig anti-Synapsin1/2, 1:1000, 106,004, Synaptic Systems, Germany). After washing, the sections were incubated with corresponding secondary antibodies: Alexa 488-conjugated goat anti-mouse IgG (1:1000, A21121, Invitrogen, USA), Alexa 568-conjugated goat anti-guinea pig IgG (1:1000, A11075, Invitrogen, USA), Alexa 568-conjugated goat anti-chicken IgG (1:1000, A11041, Invitrogen, USA) or Alexa 647-conjugated goat anti-rabbit IgG (1:1000, A32733, Invitrogen, USA) for 2 h at 37 °C temperature; then mounted with DAPI solution after the final wash. The sections were imaged by a laser scanning spectral confocal microscope (TCS SP8, Leica Microsystems, USA).

### Analysis of glia morphology and interaction

Confocal images of microglia (Iba1) and astrocytes (Gfap) were collected with Z-stack (optical slices of 1 μm) using 20 × objective. Images were acquired using a 2 airy unit (AU) pinhole while holding constant the gain and offset parameters for all sections and rats per experiment. Images were taken from three animals in each group. Three sections from each animal were examined. The number of glia was counted stereologically [38].

To quantify the morphology of the glial cell, Iba1-positive microglia were imaged under a 63 × oil objective using the confocal microscope at a step size of 0.5 μm. The image background was subtracted and microglia morphology was analyzed using the AnalyzeSkeleton in ImageJ (NIH, USA) [39]. Images were taken from three animals in each group and three sections from each animal for microglia counting. At least 12 randomly chosen microglia in the CA1 per animal were analyzed.

To quantify the percentage of astrocytes contacted by microglia, Z-series stacks of confocal images of Gfap^+^ cells and Iba1^+^ cells were taken. The projection images were traced and were analyzed with ImageJ. Briefly, images were acquired at 1 μm Z-stack intervals. Gfap-positive fluorescence intensity with cell bodies contacting microglia processes or cells were counted using ImageJ-3D reconstruction with a 10 μm, 30 μm and 50 μm diameter distance from the microglia cell body, respectively. At least 10 randomly chosen Iba1^+^ cells in the CA1 per animal were analyzed.

### Synaptic puncta staining and analysis

Phagocytic microglia were identified by immunolabeling with Cd68. Colabeling of lysosomal marker Cd68 and Synapsin1/2 was used to image the successful progress from microglial phagocytosis. The intensity of Cd68^+^ and Synapsin1/2 clusters within microglia was measured using ImageJ [40]. Briefly, brain sections were co-immunostained with the anti-Synapsin1/2, anti-Iba1 and anti-Cd68 antibodies. Sections were imaged with the 63 × objective oil with 2 × zoom using the confocal microscope at 0.5 μm Z-stack step. Image background was subtracted using ImageJ, and microglia and synapsin intensity were calculated using Plot Profile in ImageJ.

Synaptic puncta were quantified as previously described. All confocal images of Synapsin1/2 were acquired with Z-stack (step at 0.5 μm) using 63 × objective with 2 × zoom. Analysis was performed using ImageJ. Images were acquired using a 1 AU pinhole while holding constant the gain and offset parameters for all sections and rats per experiment. All contrasting images were adjusted to the same threshold for the analysis. Post-analysis images were adjusted for brightness and contrast across the entire image for presentation.

### Electroencephalography (EEG) recording

**Fig. 1:**
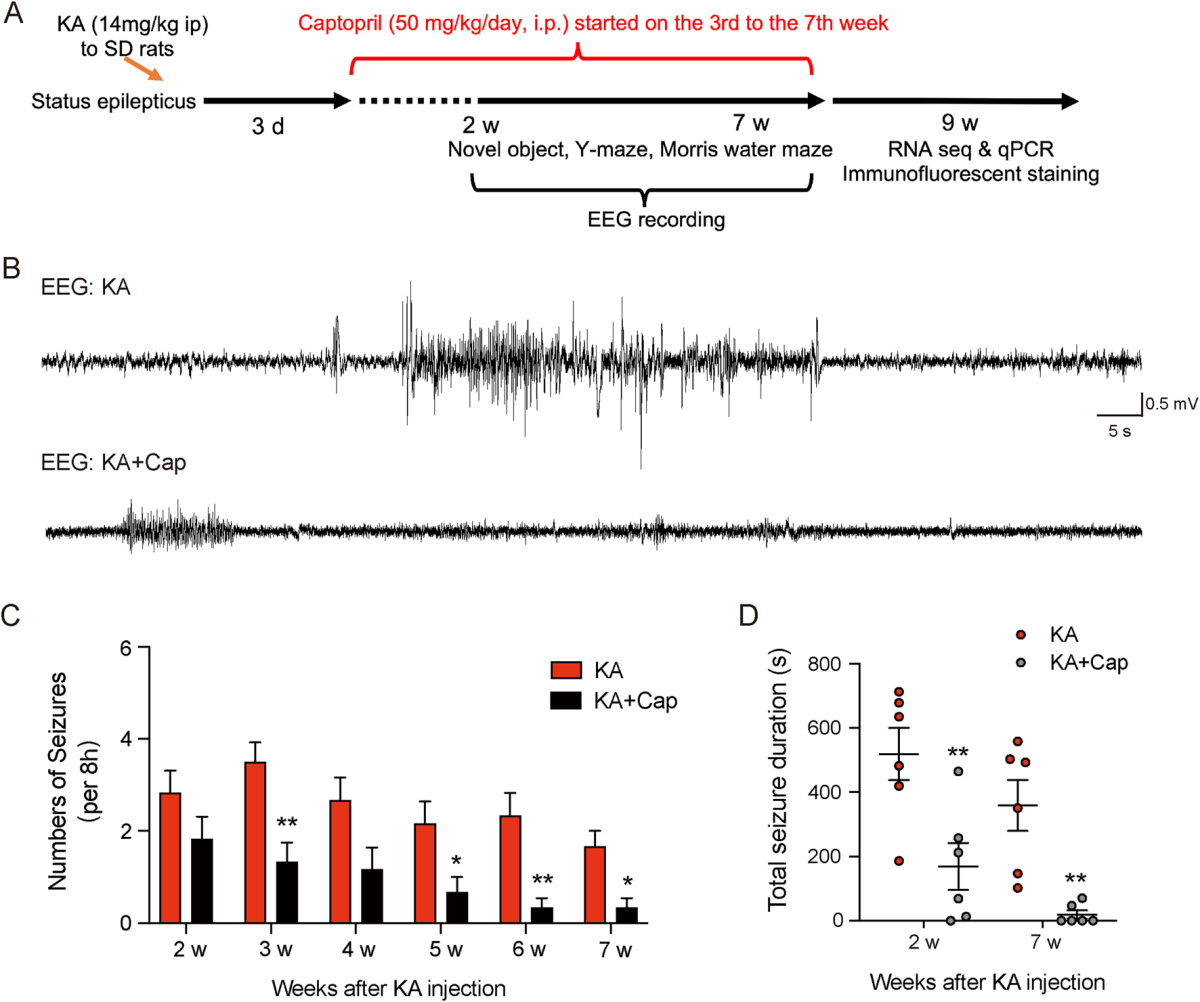
Captopril treatment attenuates the KA-induced epilepsy in rats. **A** The experimental design is shown. **B** Representative 2 min EEG recording during epilepsy (7 weeks after KA induction) indicates the captopril treatment significantly suppresses the KA-induced recurrent seizures. **C**, **D** Quantification of the frequency and total seizure duration validates the captopril treatment significantly suppresses the KA-induced recurrent seizures. *n* = 6 rats/group, *p < 0.05, **p < 0.01, Student’s *t* test. Data are presented as mean ± SE

The rats were monitored for seizures by weekly video-EEG recording sessions from week 2 to 7 after KA administration (Fig. 1A). For the surgical implantation of epidural electrodes, rats were anesthetized with 10% chloral hydrate in a stereotaxic frame 3 days after KA administration. For cortical EEG recordings, we implanted recording electrode on the left side of the rats (AP: − 4.08 mm; ML: 3.6 mm; DV: − 0.8 mm). Two screws were placed on the right and inferior sides of the cerebellum’s skull to serve as reference and ground electrodes for EEG recording. All screws were soldered on electronic pins and were secured with dental cement. After the surgery, rats were allowed for 7 days of recovery before EEG recording. Rats were acclimated in cylindrical 10-inch-diameter acrylic cages for at least 1 day before monitoring with a digital video-EEG acquisition system (Spike 2, CED, UK). EEG was acquired using standard alternating current amplifiers with no bandpass filters. Rats were monitored continuously for the 7 weeks, and EEG was synchronized with video recording continuously for 8 h/day. Video-EEG data were analyzed by two independent trained observers. All EEG data from each monitoring session were reviewed for electrographic seizures, and video was analyzed as needed to confirm behavioral correlates of electrographic seizures and to rule out sources of artifacts. Electrographic seizures were identifiable as the discrete periods of repetitive, evolving spike discharges that lasted at least 30 s. In addition, interictal spikes were identified and defined as a fast (200 ms) epileptiform waveform that was at least twice the amplitude of the background activity [41]. Seizure frequency (number of seizures per 8 h period) and total seizure duration (per 8 h period) were counted.

### Behavioral analysis

A metallic Y-maze comprising 3 arms forming the Y shape was used to estimate the short-term memory in the present investigation. Each arm was 35 cm long, 25 cm high, and 10 cm wide and was positioned at 120° extending from a central platform. Each rat was placed on the central platform and allowed to move freely through the maze for 8 min. The total number and direction of arm entries were recorded. Nonoverlapping entrance sequences were defined as spontaneous alternations. The percentage of spontaneous alternation behavior was calculated as the ratio of actual alternations to possible alternations multiplied by 100. The maze was cleaned with 70% ethanol after each rat was tested to remove any olfactory cues that may have resulted in erroneous observations.

The Novel object recognition (NOR) task is a commonly used behavioral assay to investigate various aspects of learning and memory. Rats were habituated to the environment on the morning of behavior day 1, and then they were introduced to two identical objects and allowed to explore them for 5 min. Twenty-four hours following the training trial, one of the objects was replaced with a novel object (which differed in color, shape and size), and rats were allowed a 5-min exploratory trial. The duration spent investigating each object and the discrimination index (novel/familiar) were calculated.

The Morris water maze (MWM) test records the learning capacity and visuospatial memory of animals. A large circular pool made of stainless steel (150 cm in diameter and 60 cm in height) was used in the present study. The pool was half-filled with water maintained at room temperature and divided arbitrarily into four equal quadrants with the help of two threads that were perpendicular to each other and fixed to the rim of the pool. A submerged platform (10 cm in width, 28 cm in height), painted in black, was put inside the target quadrant of this pool 2 cm below the water surface. During the four subsequent days, the rats underwent three trials per day with the platform in place. For each training trial, rats were placed in the water facing the pool wall in different pool quadrants, with an interval of at least 30 min between the trials. The maximum time for each trial was 60 s. When a rat located the platform, it was permitted to remain on it for 10 s. If the rat did not locate the platform within 60 s, it was gently guided onto the platform and was placed on the platform for 10 s. The mean escape latency (MEL) time taken by each rat to find the hidden platform was recorded for each rat during each of the trials performed over the four testing days and was used as an index of acquisition or learning. On the fifth day, the platform was removed from the pool and rats were allowed to swim for 60 s to search for the previously acquired information. The time spent by each rat in the target quadrant in which the hidden platform was previously placed, was recorded as the escape latency.

### Statistical analysis

GraphPad Prism 8.0 software (GraphPad Software Inc, USA) was used for graphing and analysis. Two-way ANOVA with Dunnett’s post hoc test was used to determine significance between time curves of different groups in the MWM test. Student’s t test was used to compare differences between two groups, and one-way ANOVA with Tukey’s post hoc test was used for multiple groups. Data in this study are presented as mean ± standard error (SE). Each experiment was performed with at least 3 biological replicates, and *p* < 0.05 was considered statistically significant.

## Results

### Captopril treatment prevents kainate-induced epilepsy

We established a KA (ip injection of 14 mg/kg KA) induced chronic epilepsy model to evaluate the effects of captopril on preventing the development of epilepsy. Captopril at a dose of 50 mg/kg/day was administered intraperitoneally from the 3^rd^ day after the KA induction (Fig. 1A). Electroencephalogram (EEG) was recorded from week 2 to 7, and the typical records of seizures in KA and KA + Cap groups in the 7^th^ week are shown in Fig. 1B. The EEG results indicated that 50 mg/kg captopril treatment significantly reduced the frequency of recurrent seizures from weeks 2 to 7, as shown in Fig. 1C (*p < 0.05 and **p < 0.01, with an average of 0.94 vs. 2.53, KA + Cap vs. KA group). Furthermore, captopril treatment reduced the seizure duration in both 2 and 7 weeks following the KA induction (2 weeks: **p < 0.01, 169.40 ± 73.07 vs. 519.00 ± 81.34; 7 weeks: *p < 0.01, 19.50 ± 12.69 vs. 337.00 ± 93.10, KA + Cap vs. KA group, Fig. 1D).

### Captopril treatment ameliorates the epilepsy-associated cognitive impairment

**Fig. 2:**
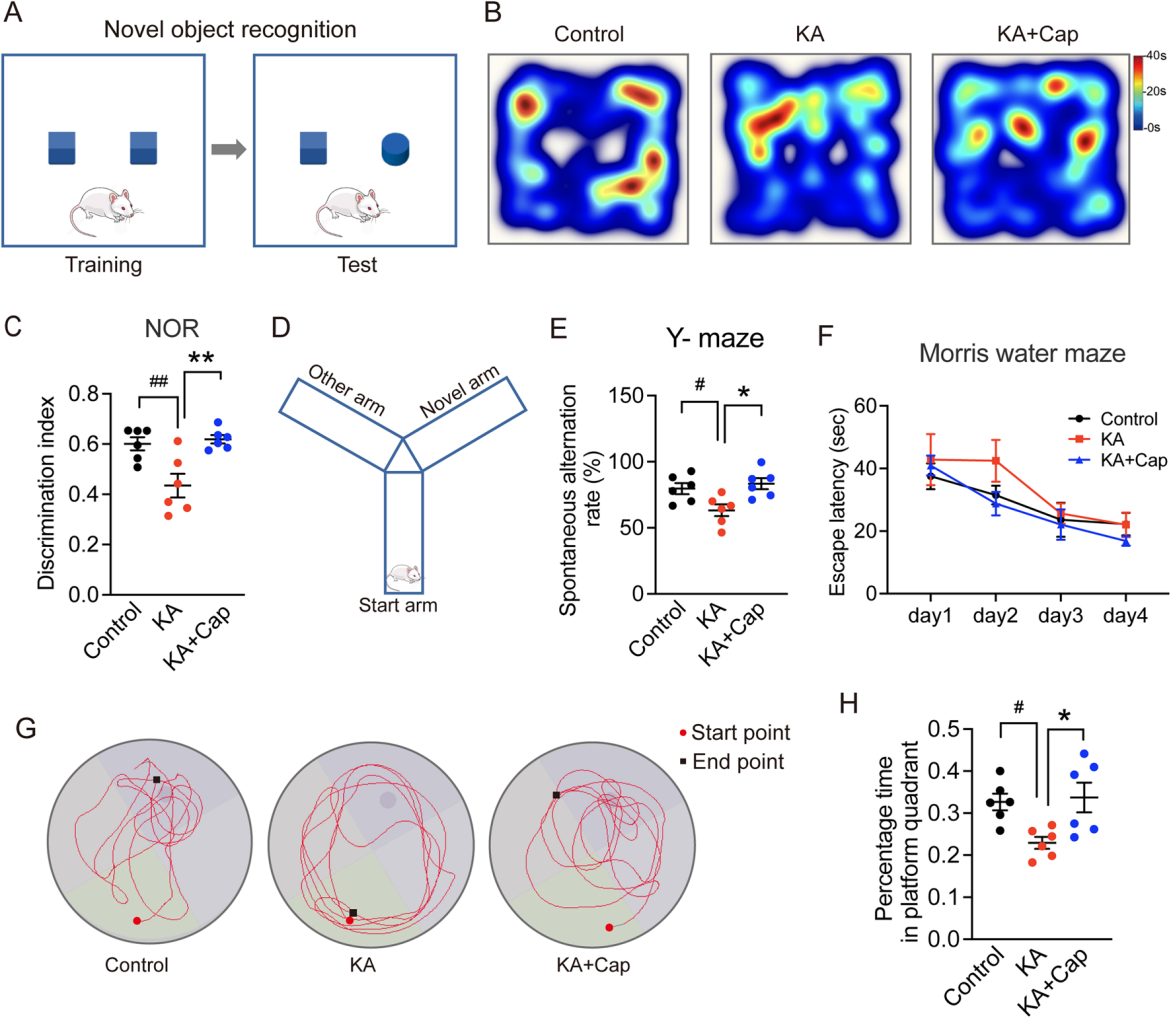
Captopril treatment ameliorates epilepsy-related memory impairment in rats. **A** The experiment diagram of NOR is shown. **B**, **C** Representative heat map of traveling track diagram around objects and quantification of discrimination index show captopril treatment significantly ameliorates the epilepsy-related cognitive function impairment. **D** A schematic diagram of Y-maze test is shown. **E** Quantification of spontaneous alteration rate in Y-maze test shows captopril treatment reverses the epilepsy-related deficits in spatial memory. **F** The mean escape latency to the hidden platform in the water maze as a short-term learning function of 4 training days shows no significant difference among the groups. *n* = 6 rats/group, two-way ANOVA followed by Dunnett’s post hoc test. **G**, **H** Representative swimming paths and quantification of time spent in the platform quadrant on day 5 (spatial probe test day) show captopril treatment significantly improves the epilepsy-impaired short-term memory. *n* = 6 rats/group, ^#^*p* < 0.05, ^##^*p* < 0.01, compared to control; *p < 0.05, **p < 0.01, compared to KA, one-way ANOVA followed by Tukey’s post hoc test. Data are presented as mean ± SE

To determine the effects of captopril on the epilepsy-associated cognitive deficits, we assessed the hippocampal-dependent short-term learning and memory performance by Y-maze and NOR test (Fig. 2A, D). The KA group exhibited memory deficits in Y-maze and NOR test, compared to the control group, which were evident from the significant reduction of the ratio of discrimination index in the NOR test (^##^*p* < 0.01, 0.43 ± 0.04 vs. 0.60 ± 0.02, KA vs. control group, Fig. 2B, C) and the alternation score in the Y-maze test (^#^*p* < 0.05, 63.22 ± 4.46% vs. 79.65 ± 4.16%, KA vs. control group, Fig. 2D, E). Moreover, captopril treatment markedly improved the ratio of discrimination index to the control level in the NOR test (**p < 0.01, 0.61 ± 0.02 vs. 0.43 ± 0.04, KA + Cap vs. KA group, Fig. 2B, C) and the alternation score in the Y-maze test, compared to the KA group (*p < 0.05, 83.35 ± 4.20% vs. 63.22 ± 4.46%, KA + Cap vs. KA group, Fig. 2D, E). Thus, captopril attenuated the KA-induced impairment in short-term memory.

Spatial learning and memory were tested using MWM 7 weeks after the KA induction. An average of three trials held each day for each group was recorded. The time escape latency in control, KA and KA + Cap groups were decreased over successive days, which showed no obvious difference on the training day 1–4 (control: 28.71 ± 3.57 s; KA: 33.22 ± 5.48 s; KA + Cap: 27.15 ± 5.20 s, Fig. 2F). On the probe test day, KA group revealed a significant decrease in the time spent in the target quadrant, where the platform was previously located, compared to the control group. Typical swimming route paths for each group are shown in Fig. 2G. The KA + Cap group spent more time in the target quadrant compared to the KA group (^#^p < 0.05, 0.23 ± 0.01 s vs. 0.33 ± 0.04 s, KA vs. control group; *p < 0.05, 0.33 ± 0.03 s vs. 0.23 ± 0.01 s, KA + Cap vs. KA group, Fig. 2H). These data indicate that the KA-induced cognitive decline is ameliorated by captopril treatment.

### Captopril treatment suppresses phagocytosis and inflammatory responses in the hippocampus 9 weeks after the KA induction

**Fig. 3:**
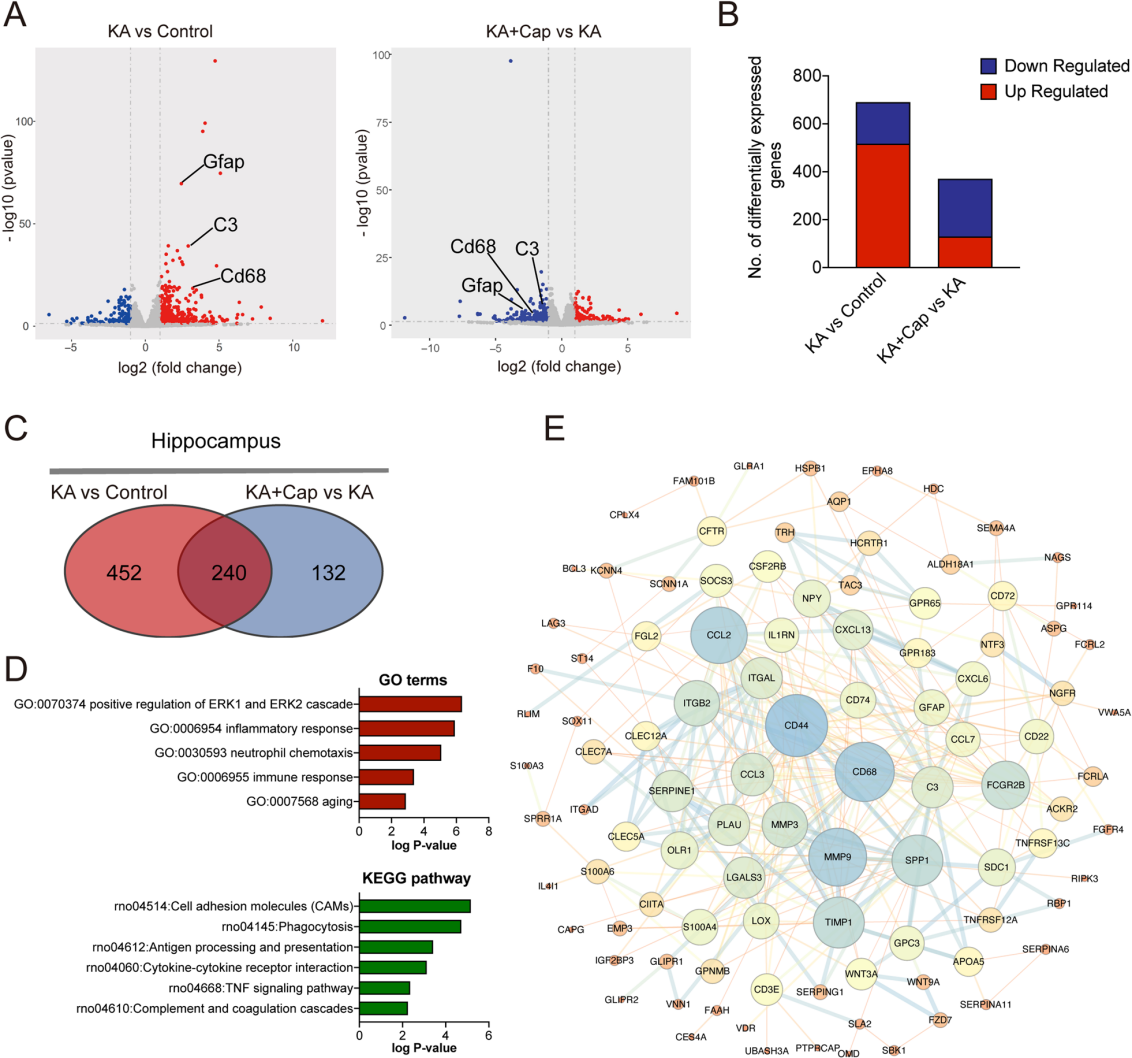
Captopril treatment reduces the KA-induced phagocytosis and inflammatory responses in the hippocampus. RNA-seq analysis was performed using the hippocampal tissues 9 weeks after the KA induction. Results were normalized to the control group. P-values were obtained by edgeR using an exact test through the negative binomial distribution. **A** The volcano plot is shown for gene expression in the hippocampus with significantly increased (red) or decreased (blue) expression (*p* < 0.05, |log2|> 1) compared between the control and KA group (left) or between the KA and KA + Cap group (right). **B** Hierarchical clustering was performed for all the significantly altered gene expression. Magenta indicates the number of up-regulated genes and blue indicates the number of down-regulated genes. **C** The number of the shared and distinct DEGs in the hippocampus in the KA and KA + Cap groups. **D** GO and KEGG analysis of the transcripts with the shared DEGs in the KA and KA + Cap groups are shown. **E** The STRING analysis of the interactions of 240 top-regulated genes recovered after captopril treatment is shown. *n* = 3 rats/group

To gain a deeper understanding of the molecular mechanisms underlying the effects of captopril treatment, we performed RNA sequencing (RNA-seq) analysis using the hippocampal tissues from the control, KA and KA + Cap groups 9 weeks after the KA induction. We identified 692 differentially expressed genes (DEGs, adjusted *p* < 0.05, 517 up and 175 down) in the KA group, compared to the control group, and 371 DEGs (130 up and 241 down) in the KA + Cap group, compared to the KA group (Fig. 3A, B). Further cross-genotype comparisons showed that captopril treatment reversed about one-third of the DEGs compared to the KA and control group (Fig. 3C). Gene Ontology (GO) and Kyoto Encyclopedia of Genes and Genomes (KEGG) pathways analysis of the DEGs revealed a significant down-regulation of various immune pathways in the KA + Cap group, including inflammatory response, immune response, phagocytosis, complement and coagulation cascades, etc. (Fig. 3D). To explore potential mechanisms related to the effects of captopril treatment, we analyzed the known and predicted protein interactions of these different genes. The results showed that the central regulatory proteins of these genes include Cd68, Cd44, Ccl2, matrix metallopeptidase 9 (Mmp9) and tissue inhibitor of metalloproteinases 1 (Timp1), all of which are associated with the glia activation-related immune and inflammatory responses (Fig. 3E).

**Fig. 4:**
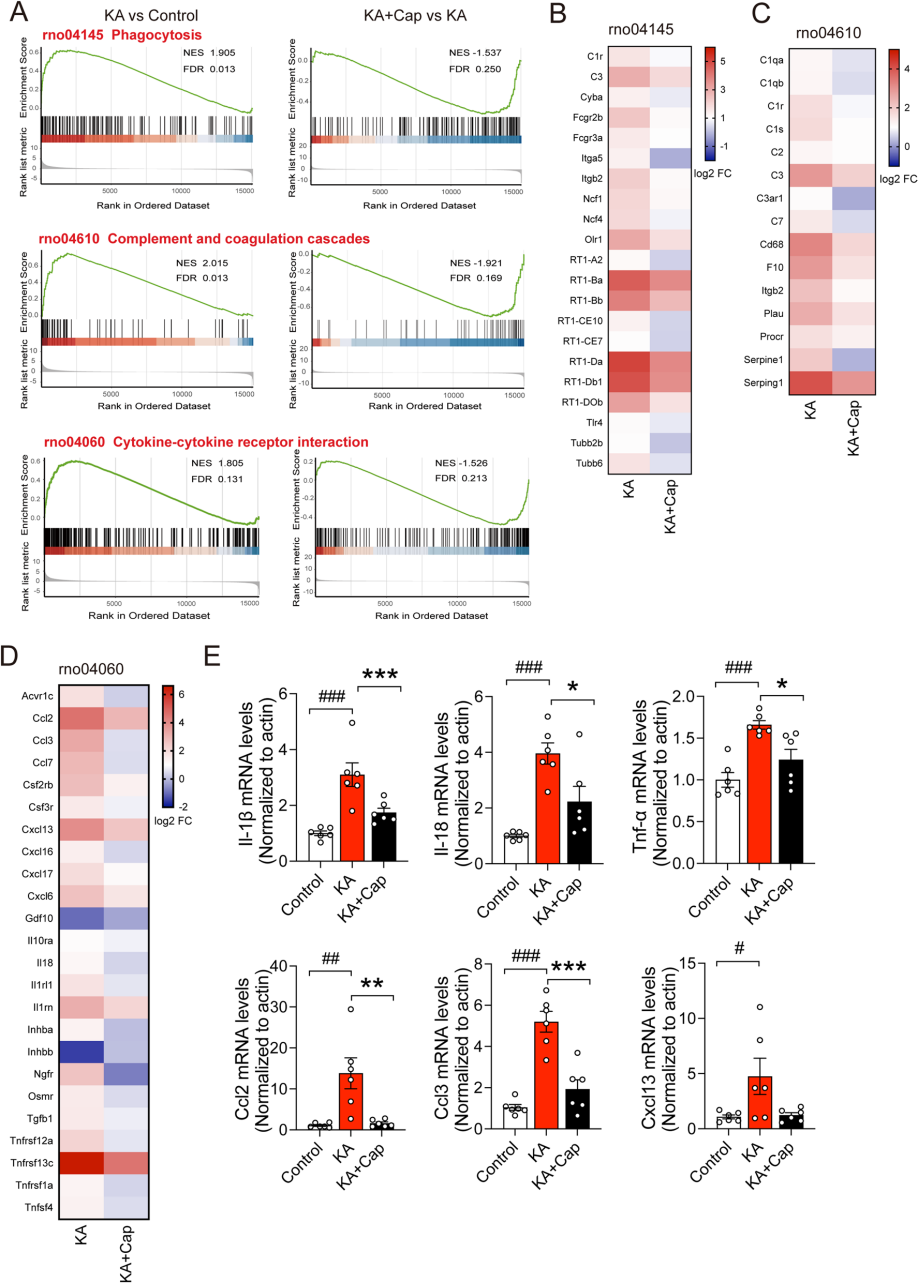
Captopril treatment suppresses the genes expression within the inflammation and phagocytosis. **A** Gene set enrichment analysis (GSEA) indicates captopril treatment recovers the KA-induced down-regulation of gene expression regarding cytokine–cytokine receptor interaction, phagocytosis, complement and coagulation cascades in the hippocampus. **B**–**D** The heat map shows the top-regulated gene expression of phagocytosis, complement and coagulation cascades, and cytokine–cytokine receptor interaction in the hippocampus in the KA and KA + Cap groups. **E** Captopril treatment significantly reduces the gene expression related to inflammatory and immunological regulation in the hippocampus compared to the KA group. n = 6 rats/group, ^#^*p* < 0.05, ^##^*p* < 0.01, ^###^*p* < 0.001, compared to control; *p < 0.05, **p < 0.01, ***p < 0.001, compared to KA, one-way ANOVA followed by Tukey’s post hoc test. Data are presented as mean ± SE

To investigate the enrichment of DEGs between the KA and KA + Cap group, we performed GSEA assay, a robust computational method that determines whether an a priori-defined set of genes shows statistical significance, concordant difference between the 2 groups. In the KA group, the genes related to the phagocytosis signaling pathway (rno 04145), the complement system (rno 04610) and cytokine–cytokine receptor interaction (rno 04060) were up-regulated compared to the control group (NES = 1.90, FDR = 0.01; NES = 2.01, FDR = 0.01; NES = 1.80, FDR = 0.13), which were significantly down-regulated by captopril treatment (NES = − 1.53, FDR = 0.25; NES = − 1.92, FDR = 0.16; NES = − 1.52, FDR = 0.21). Noticeably, the changes in phagocytosis were more significant than that of inflammatory cytokines (Fig. 4A).

Most significantly down-regulated genes by captopril treatment were immune response-related genes. These DEGs can be divided explicitly into phagocytosis related genes, such as C1r, C3, Itga5, Itgβ2, etc. (Fig. 4B); complement system signaling pathway genes, such as C1q, C3, C3ar1, Cd68, etc. (Fig. 4C) and chemokine genes, such as Ccl2, Ccl3, Ccl7, Cxcl13, etc.; cytokine genes, such as Il-18, Il-1β and cytokine receptor genes, such as Il-1r, etc. (Fig. 4D).

To confirm the reliability of the expression profiles generated by the RNA-seq and DEGs analysis, qRT-PCR was performed to validate the expression of the typical significantly up-regulated genes. Microglia and astrocytes activation is associated with increased proinflammatory cytokines, including Il-1β, Il-18 and Tnf-α, and chemokines, such as Ccl2, Ccl3 and Cxcl13, all of which were elevated in the hippocampus of the KA group. Captopril treatment significantly lowered the KA-induced transcriptional levels of cytokine, consistent with the results from the RNA-seq experiments (Il-1β: ***p < 0.001, 1.74 ± 0.15 vs. 3.09 ± 0.42; Il-18: *p < 0.05, 2.23 ± 0.54 vs. 3.95 ± 0.38; Tnf-α: *p < 0.05, 1.24 ± 0.12 vs. 1.66 ± 0.05; Ccl2: **p < 0.01, 1.61 ± 0.34 vs. 13.80 ± 3.77; Ccl3: ***p < 0.001, 1.93 ± 0.45 vs. 5.19 ± 0.50, KA + Cap vs. KA group, Fig. 4E).

### Captopril treatment reduces the activation of astrocytes and microglia in the hippocampus 9 weeks after the KA induction

**Fig. 5:**
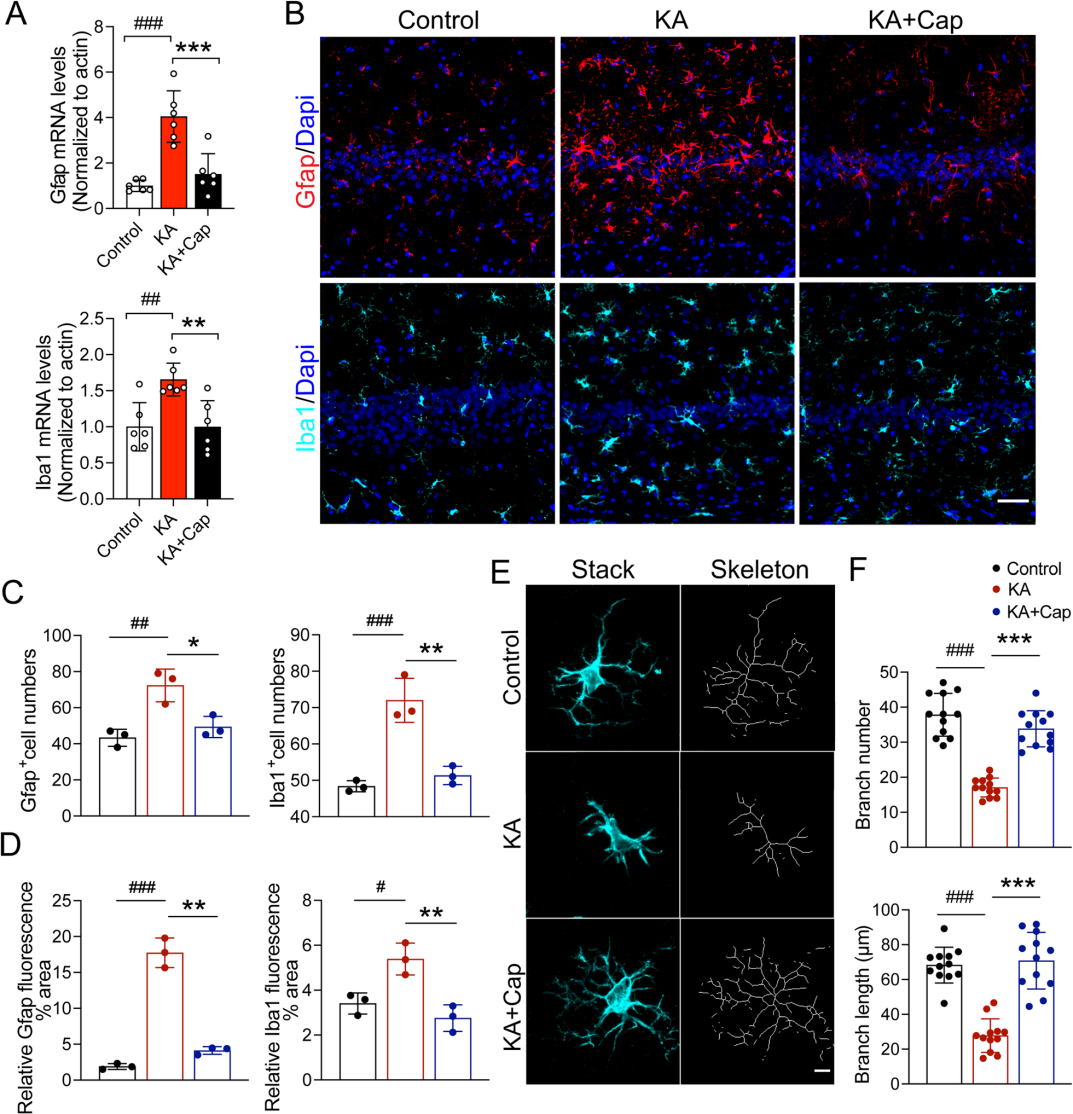
Captopril treatment suppresses the KA-induced glial cell activation in the hippocampus. **A** Captopril treatment significantly reduced the KA-induced gene expression of glial markers in the hippocampus 9 weeks after the induction. *n* = 6 rats/group. **B** Representative images show Iba1 (cyan) and Gfap (red) staining in the control, KA and KA + Cap groups. Scale bar = 50 μm. **C** Quantitative analysis shows that captopril treatment significantly attenuates the KA-induced upregulation of the number of Iba1 (cyan) and Gfap (red) positive glial cells in the hippocampal CA1. **D** Quantitative analysis shows captopril treatment significantly attenuates the KA-induced upregulation of Iba1 (cyan) and Gfap (red) intensity in the hippocampal CA1, *n* = 3 images from 3 rats/group. **E** Representative images show the microglial morphology (Iba1, cyan; transformed skeleton, gray) in the control, KA and KA + Cap groups. Scale bar = 5 μm. **F** Quantification of the branch number (top) and length (bottom) of the microglia process shows that captopril treatment significantly attenuates the epilepsy-induced microglia activation compared to the KA group. *n* = 12 cells from 3 rats/group, ^#^*p* < 0.05, ^##^*p* < 0.01, ^###^*p* < 0.001, compared to control; *p < 0.05, **p < 0.01, ***p < 0.001, compared to KA, one-way ANOVA followed by Tukey’s post hoc test. Data are expressed as mean ± SE

Given that microglia and astrocytes are the main cell types exerting inflammatory and immune responses in the brain, we performed qRT-PCR and immunofluorescence staining for microglia and astrocyte marker genes to investigate the effects of captopril on glial activation in the hippocampus 9 weeks after the KA induction when recurrent seizures were supposedly formed. We found that the mRNA expression of Iba1 and Gfap was significantly increased in the hippocampus in the KA group, which were almost totally reversed by captopril treatment (Iba1: ^##^*p* < 0.01, 1.65 ± 0.09 vs. 1.00 ± 0.13, KA vs. control group; **p < 0.01, 0.99 ± 0.14 vs. 1.65 ± 0.09, KA + Cap vs. KA group; Gfap: ^###^*p* < 0.001, 4.04 ± 0.46 vs. 1.00 ± 0.10, KA vs. control group; ***p < 0.001, 1.51 ± 0.36 vs. 4.04 ± 0.46, KA + Cap vs. KA group, Fig. 5A). The quantitative analysis of immunostaining in the hippocampal CA1 area revealed that captopril treatment markedly decreased GFAP^+^ and Iba1^+^ cell numbers and area compared to the KA group, which was consistent with the results from the qRT-PCR analysis. (Gfap: *p < 0.05, 49.33 ± 3.38 vs. 72.33 ± 5.23; **p < 0.01, 4.11 ± 0.30 vs. 17.74 ± 1.19, KA + Cap vs. KA group; Iba1: **p < 0.01, 51.33 ± 1.45 vs. 72.00 ± 3.51; **p < 0.01, 2.75 ± 0.34 vs. 5.40 ± 0.41, KA + Cap vs. KA group, respectively, Fig. 5B–D).

In addition, we found that microglia in the KA group underwent a morphological change from a “resting” ramified phenotype to an “activated” bushy phenotype. Sholl analysis of astrocyte microglia morphologies using the ImageJ software showed reduced branch number and branch length in the KA group. Noticeably, these phenotypes were reversed by captopril treatment (***p < 0.001, 33.83 ± 1.49 vs. 17.08 ± 0.77; 70.80 ± 4.69 vs. 27.78 ± 2.78 KA + Cap vs. KA group, Fig. 5E, F).

**Fig. 6:**
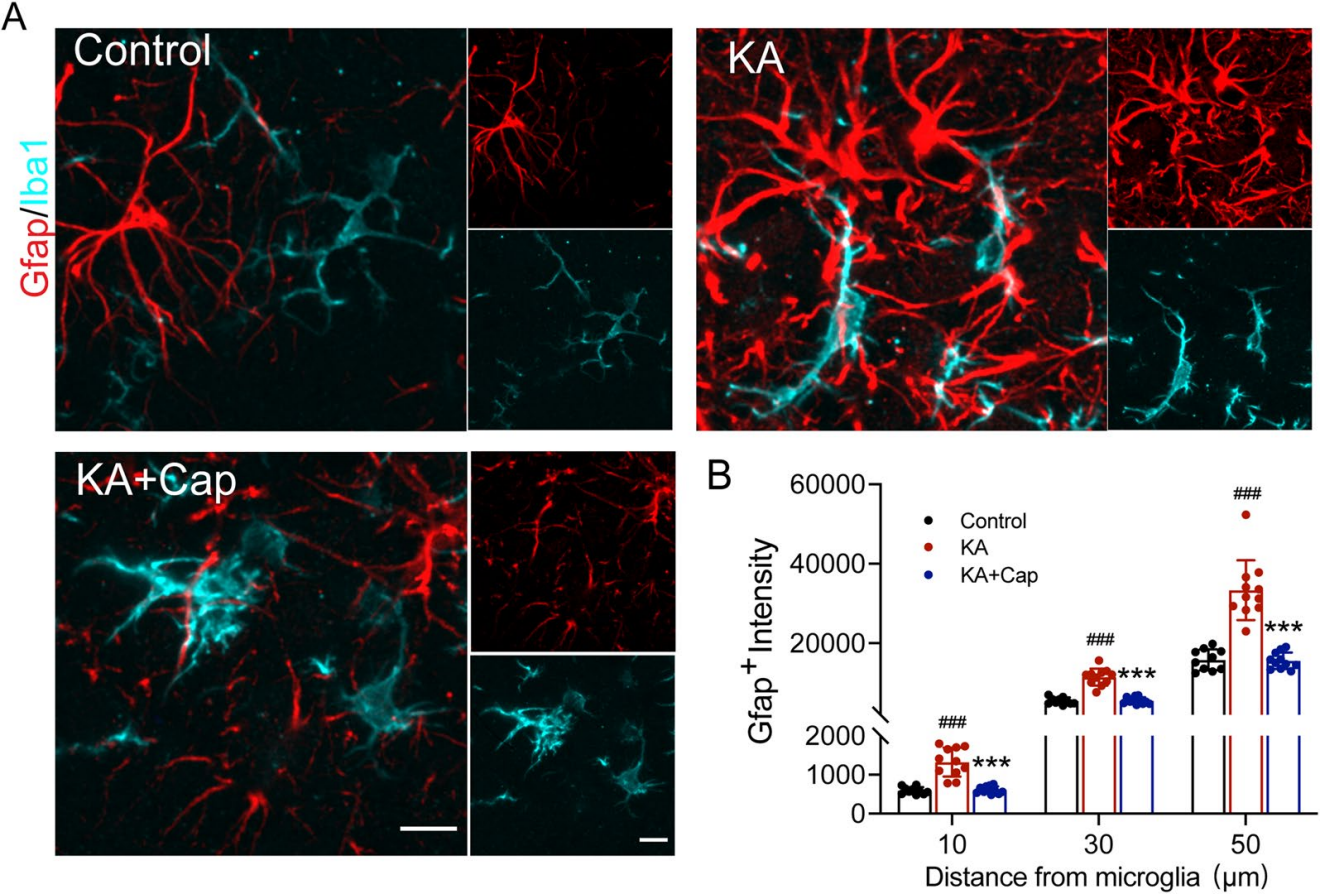
Captopril treatment suppresses the KA-induced contact between glial cells in the hippocampus. **A** Representative anti-Iba1 and anti-Gfap double immunostaining indicates that captopril treatment reduces the KA-induced contact between astrocytes and microglia. Scale bar = 5 μm. **B** Quantification of Gfap (red) signal intensity around microglia (cyan) is shown. *n* = 10–12 cells from 3 rats/group, ^###^*p* < 0.001, compared to control; ^***^p < 0.001, compared to KA, one-way ANOVA followed by Tukey’s post hoc test. Data are expressed as mean ± SE

Double immunostaining of astrocytes and microglia showed that they had a tendency to approach each other in the KA group, indicated by the fluorescence intensity of Gfap at different distances of 10, 30 and 50 μm from the center of microglia, which was significantly restored by the captopril treatment (10 μm: ***p < 0.001, 608.10 ± 21.17 vs. 1323.00 ± 111.70; 30 μm: ***p < 0.001, 5570.00 ± 254.30 vs. 11,513.00 ± 637.5; 50 μm: ***p < 0.001, 15,584.00 ± 619.8 vs. 33,367.00 ± 2269.00, KA + Cap vs. KA group, Fig. 6A, B). Taken together, these data suggest a pivotal role for captopril in ameliorating the KA-induced glial cell activation and contact in the hippocampus.

### ***Captopril treatment attenuates the KA-induced glial activation through ***C3–C3ar*** negative expression in the hippocampus 9 weeks after the KA induction***

**Fig. 7:**
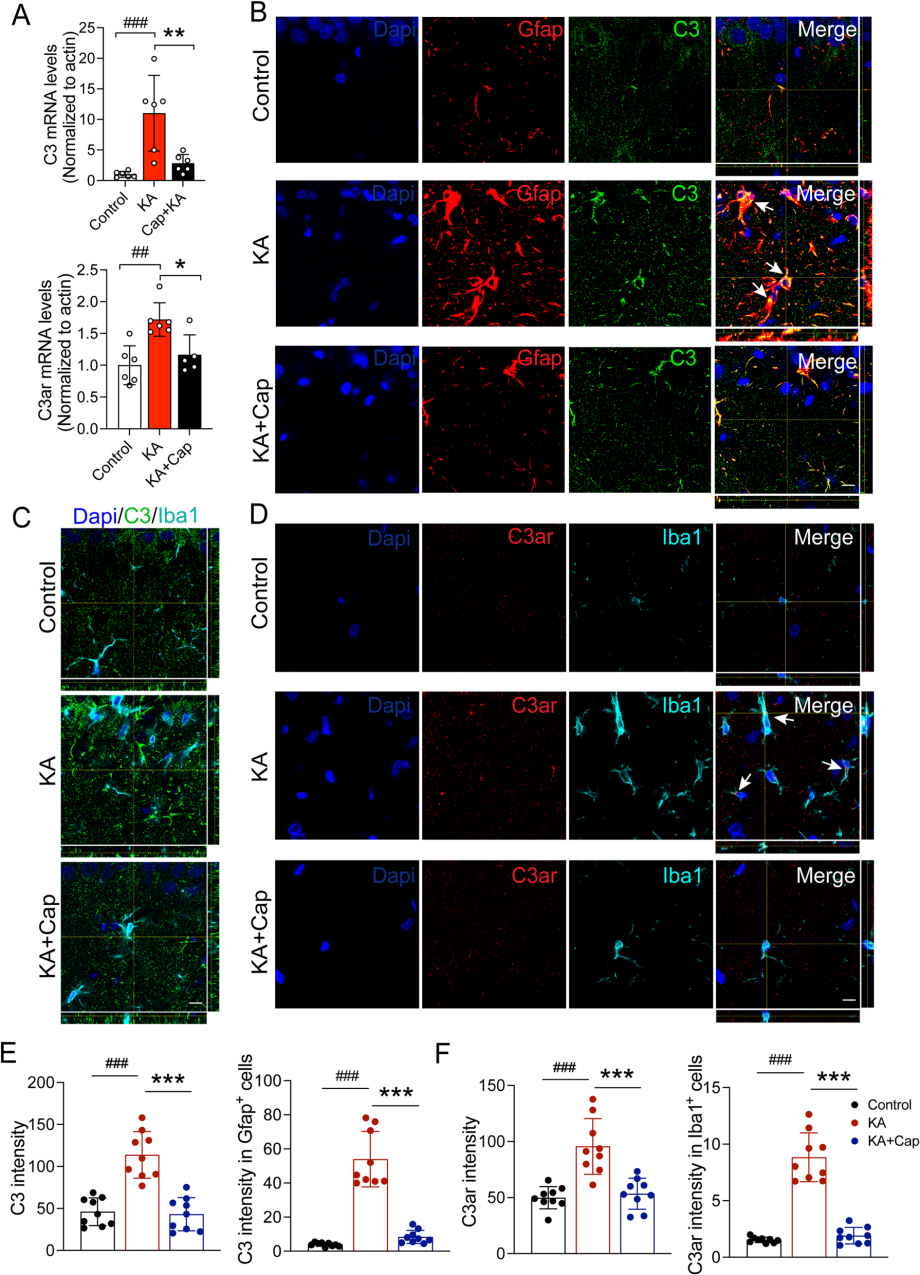
Captopril treatment attenuates the KA-induced C3 expression in astrocytes and C3ar expression in microglia in the hippocampus 9 weeks after the KA induction. **A** Captopril significantly reduces the KA-induced upregulation of mRNA expression of C3 and C3ar in the hippocampus. *n* = 6 rats/group. **B** Representative anti-C3 (green) and anti-Gfap (red) double immunostaining indicates captopril attenuates the KA-induced elevation in astrocytic C3 production. **C** Representative anti-C3 (green) and anti-Iba1 (cyan) double immunostaining indicates C3 is not expressed in microglia in the hippocampus. **D** Representative anti-C3ar (red) and anti-Iba1 (cyan) double immunostaining indicates captopril attenuates the KA-induced elevation in microglia C3ar expression. **E** Quantification of C3 (green) signal intensity within Gfap^+^ (red) astrocytes is shown. **F** Quantification of C3ar (red) signal intensity within Iba1 (cyan) microglia is shown. Scale bar = 10 μm. *n* = 9 images from 3 rats/group, ^##^*p* < 0.01, ^###^*p* < 0.001, compared to control; ^*^p < 0.05, **p < 0.01; ***p < 0.001, compared to KA, one-way ANOVA followed by Tukey’s post hoc test. Data are expressed as mean ± SE

To investigate the role of C3–C3ar signaling in immune regulation and glial activation, we examined the mRNA expression of C3 and C3ar in the hippocampus 9 weeks after the KA induction. The results of RT-qPCR showed that mRNA expression of C3 and C3ar was increased in the KA group, which was almost completely abolished by captopril treatment (C3: ^###^*p* < 0.001, 11.02 ± 2.53 vs. 1.00 ± 0.19 KA vs. control group; **p < 0.01, 2.81 ± 0.59 vs.11.02 ± 2.53, KA + Cap vs. KA group; C3ar: ^##^*p* < 0.01, 1.71 ± 0.11 vs. 1.00 ± 0.67, KA vs. control group; *p < 0.05, 1.16 ± 0.14 vs. 1.71 ± 0.11, KA + Cap vs. KA group, Fig. 7A).

Consistent with the elevated C3 and C3ar mRNA in the hippocampus, we detected a drastic increase of C3 intensity in the CA1, which was concentrated in the Gfap^+^ astrocytes in the KA group (Fig. 7B). In addition, the C3ar intensity in the CA1 area was significantly increased, concentrated in Iba1^+^ microglia (Fig. 7D). Noticeably, both C3 and C3ar co-localized signals with astrocytes or microglia were significantly attenuated after captopril treatment (***p < 0.001, C3 intensity: 43.16 ± 6.56 vs.113.80 ± 9.25; C3 in Gfap: 8.38 ± 1.28 vs. 53.98 ± 5.42; C3ar intensity: ***p < 0.01, 54.43 ± 4.60 vs. 96.64 ± 8.27; C3ar in Iba1: 1.91 ± 0.24 vs. 8.82 ± 0.71, KA + Cap vs. KA group, Fig. 7E, F). Co-immunostaining of C3 and Iba1 revealed no positive C3 protein signal in Iba1-positive microglia (Fig. 7 C). These results suggest that captopril attenuates epilepsy-related astrocyte and microglia activation and the production of proinflammatory cytokines through the C3–C3ar signaling pathway.

### Captopril treatment reduces microglia-mediated synaptic phagocytosis in the hippocampus 9 weeks after the KA induction

**Fig. 8:**
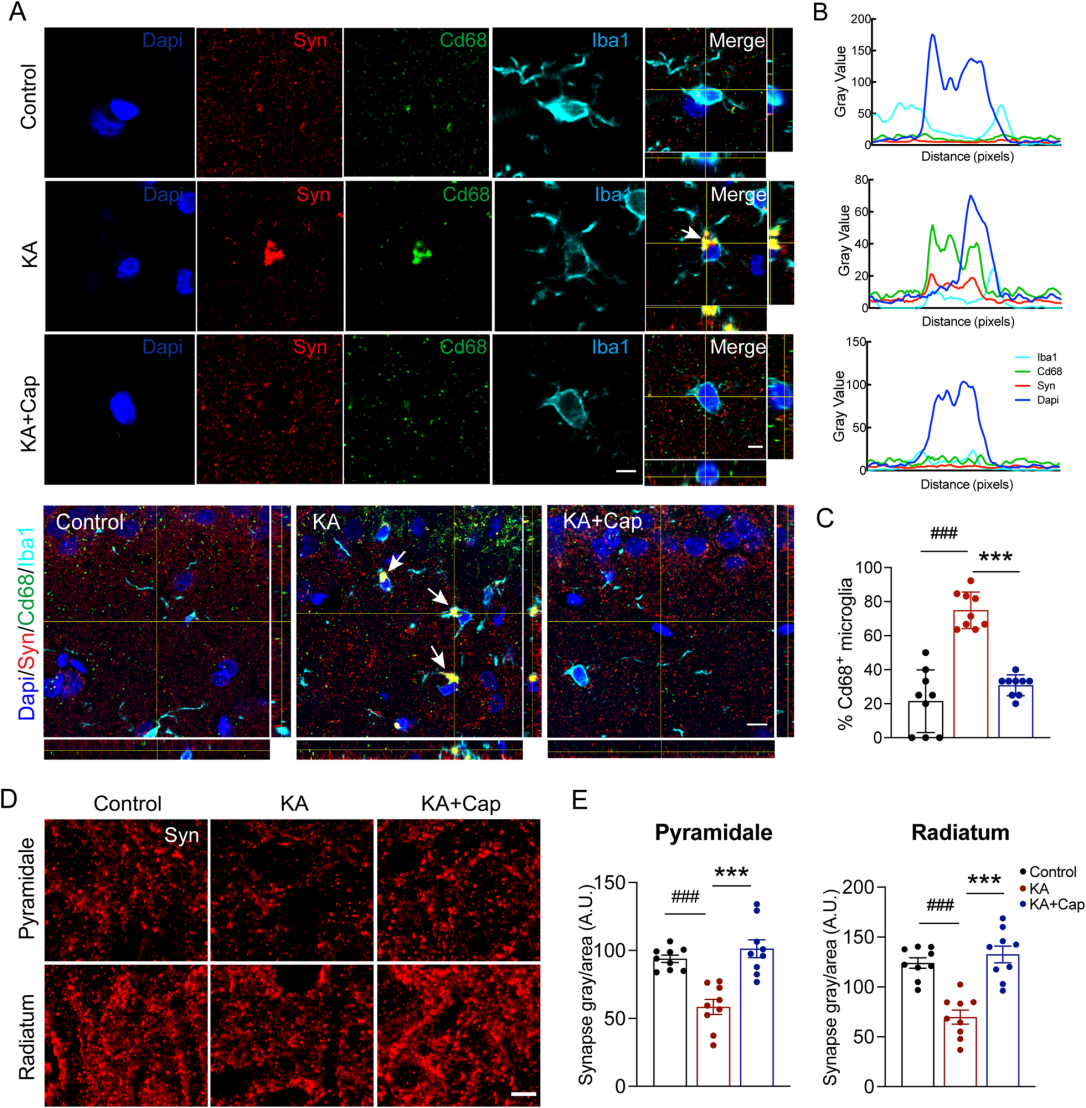
Captopril treatment reduces the KA-induced synaptic phagocytosis by activated microglia. **A** Immunostaining of Cd68 (green), synapsin (red) and Iba1 (cyan) in the hippocampal CA1 indicates captopril significantly attenuates the KA-induced synaptic pruning by microglia. Top, scale bar = 5 μm. Bottom, scale bar = 10 μm. **B** Quantification of Cd68 and synapsin immunofluorescent signals within Iba1^+^ microglia indicates captopril treatment significantly reduces the KA-induced microglia engulfment capacity as measured by lysosomal content within each microglia (CD68 immunoreactivity per cell). **C** Quantification shows that captopril reduces the KA-induced upregulation of the proportion of microglia with Cd68^+^ (green) signal in the hippocampal CA1. **D** Representative confocal images of synapsin (red) from pyramidale and radiatum in the hippocampal CA1 are shown. Scale bar = 5 μm. **E** Quantification of punctuated integrated signal intensity of synapsin indicates captopril significantly attenuates the KA-induced synaptic loss in the pyramidale and radiatum layer of hippocampal CA1. Scale bar = 5 μm. n = 9 images from 3 rats/group, ^###^*p* < 0.001, compared to control; ***p < 0.001, compared to KA, one-way ANOVA followed by Tukey’s post hoc test. Data are presented as mean ± SE

The complement pathway has been implicated in microglia-mediated synapse pruning. The activation of C3 receptors on microglia triggers their activation and synaptic elimination by phagocytosis [42]. To further characterize the effects of captopril treatment on microglia phenotype, Cd68 and Iba1 double staining were conducted to identify phagocytic microglia (Fig. 8A). Quantification of Cd68 immunoreactivity revealed that captopril treatment dampened the proportion of Cd68-positive microglia in the hippocampal CA1 area (***p < 0.001, 21.48 ± 6.13% vs. 74.90 ± 3.57%, KA + Cap vs. KA group; ^###^*p* < 0.001, 74.90 ± 3.57% vs. 30.74 ± 2.03%, KA vs. control group, respectively, Fig. 8B, C). To investigate whether captopril treatment attenuated total synaptic loss in the model, we quantified the synapsin immunoreactivity in the hippocampal CA1 (Fig. 8D), where it showed a significant reduction of synapsin positive signals in the hippocampal CA1 area in the KA group, compared to the control group (^###^*p* < 0.001, pyramidale layer: 58.42 ± 5.51 vs. 93.88 ± 2.68; radiatum layer: 69.73 ± 6.96 vs. 124.00 ± 5.17, KA vs. control group, Fig. 8E). Captopril treatment significantly ameliorated the KA-induced synaptic loss indicated by an elevated synapsin immunoreactivity in both the pyramidale and radiatum layers, compared to the KA group (***p < 0.001, pyramidale layer: 101.30 ± 19.58 vs. 58.42 ± 5.51; radiatum layer: 132.70 ± 8.32 vs. 69.73 ± 6.96, KA + Cap vs. KA, Fig. 8E). Together, these results indicate that captopril treatment attenuates the KA-induced synaptic phagocytosis by microglia in the hippocampal CA1, which may further prevent synaptic loss in the KA-induced rat model of epilepsy.

### Intranasal C3a treatment leads to cognitive impairment in the KA-induced model of epilepsy after captopril treatment

**Fig. 9:**
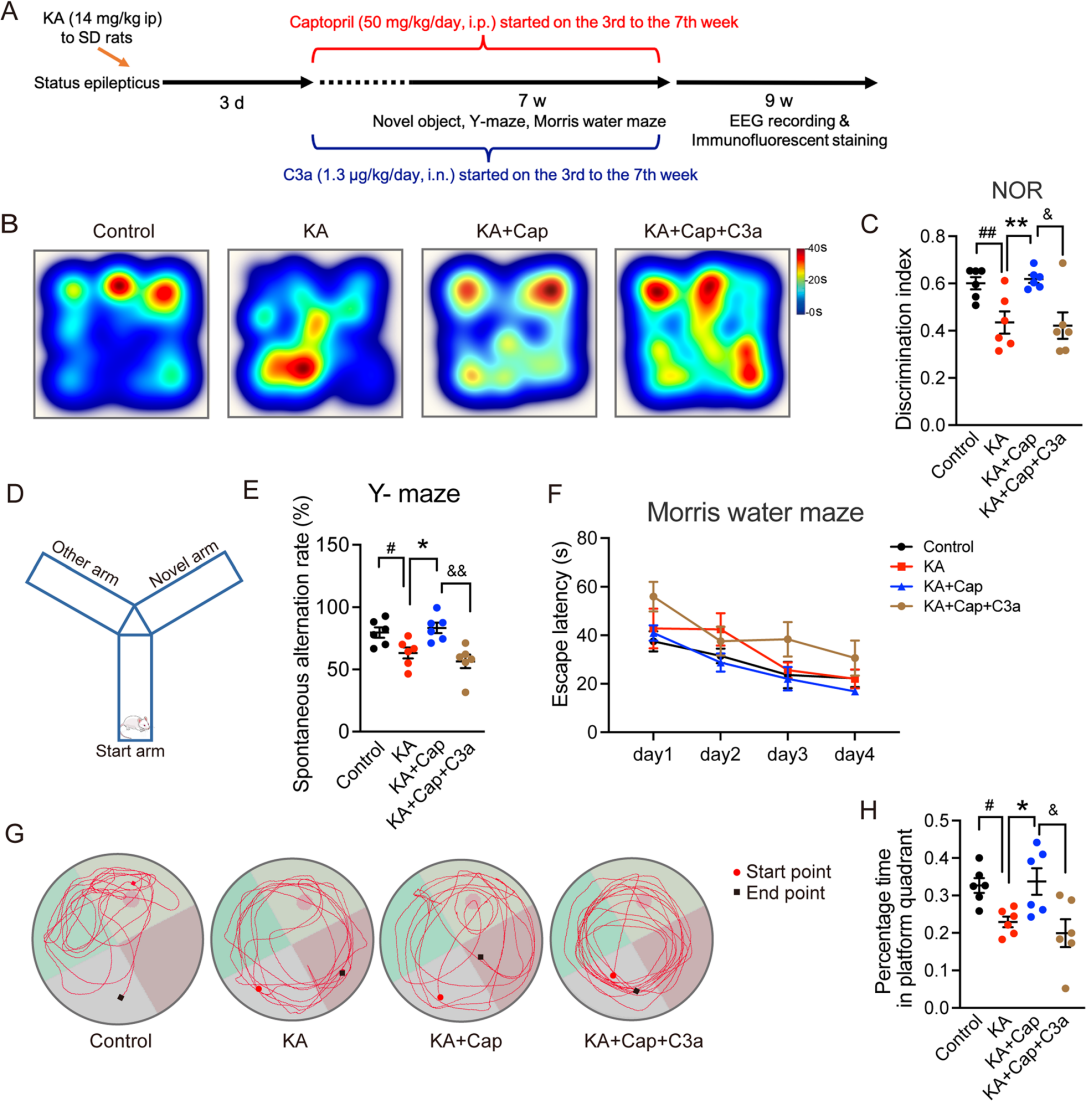
Nasal instillation of C3a aggravates epilepsy-related cognitive dysfunction after captopril treatment. **A** The experimental design is shown. **B** Representative heat maps of the duration the rat spent around the old and new objects are shown. **C** C3a nasal drip reverses the cognitive amelioration by captopril treatment in NOR test. **D** A schematic diagram of Y-maze test is shown. **E** C3a nasal drip reverses the spatial memory amelioration from captopril in spontaneous alternation in Y-maze task. **F** The mean escape latency to the hidden platform in the water maze as a short-term learning function of 4 training days shows no significant difference. *n* = 6 rats/group, two-way ANOVA followed by Dunnett’s post hoc test. **G** Representative swimming paths in the MWM test for day 5 (spatial probe test day) are shown. **H** C3a nasal drip significantly reverses the therapeutic effects of captopril on the short-term memory impairment, indicated by a reduction in the percentage of time spent in the target quadrant where the platform is located. *n* = 6 rats/group, ^#^*p* < 0.05, ^##^*p* < 0.01, compared to control; *p < 0.05, **p < 0.01, compared to KA + Cap; ^&^*p* < 0.05; ^&&^*p* < 0.01, compared to KA + Cap, one-way ANOVA followed by Tukey’s post hoc test. Data are presented as mean ± SE

The above results demonstrated a prominent role of C3–C3ar signaling in the therapeutic effects of captopril on the KA-induced model of epilepsy. To further verify that captopril treatment acted through the suppression of C3–C3ar signaling, C3a was administered intranasally to the captopril-treated rats after the KA induction, then EEG and cognition-related behavioral performance were assessed (Fig. 9A). The KA + Cap + C3a group exhibited more severe memory deficits in both NOR and Y-maze tests compared to the KA + Cap group, evident from a significant reduction of the ratio of discrimination index in NOR test (^&^*p* < 0.05, 0.42 ± 0.05 vs. 0.61 ± 0.04, KA + Cap + C3a vs. KA + Cap group, Fig. 9B, C) as well as a reduction of the alternation score in Y-maze test (^&&^*p* < 0.01, 56.46 ± 5.42% vs. 83.35 ± 4.20%, KA + Cap + C3a vs. KA + Cap group, Fig. 9D, E). The mean time escape latency of 4 groups were all reduced over successive days, which showed no obvious difference (control: 28.71 ± 3.57 s; KA: 33.22 ± 5.48 s; KA + Cap: 27.15 ± 5.20 s; KA + Cap + C3a: 40.61 ± 5.39 s, Fig. 9F). For the MWM test, typical swimming route paths for each group are shown in Fig. 9G. Furthermore, on the probe test day of the MWM task, the KA + Cap + C3a group spent less time in the target quadrant compared to the KA + Cap group (^&^*p* < 0.05, 0.20 ± 0.03 s vs. 0.33 ± 0.03 s, KA + Cap + C3a vs. KA + Cap group, Fig. 9H). Taken together, intranasal C3a worsened the cognitive deficits after captopril treatment in epileptic rats.

### Intranasal C3a treatment leads to the development of epilepsy and synaptic phagocytosis after captopril treatment

**Fig. 10:**
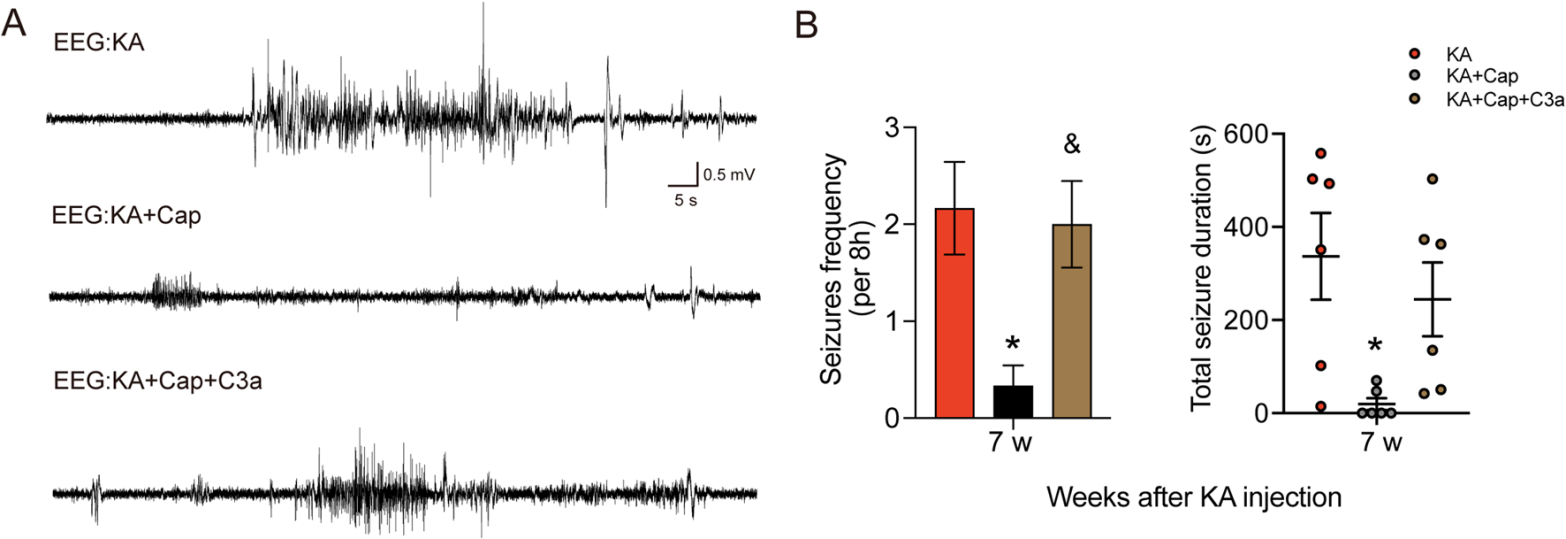
Intranasal instillation of C3a promotes epileptogenesis after captopril treatment in the KA-induced model of epilepsy. **A** Representative 2-min EEG recording during epilepsy (7 weeks after KA induction) from the KA, KA + Cap, and KA + Cap + C3a groups are shown. **B** C3a nasal drip significantly elevates the frequency and total duration of seizures compared to the KA + Cap group 7 weeks after the KA induction. *n* = 6 rats/group, *p < 0.05, compared to KA; ^&^*p* < 0.05, compared to KA + Cap, one-way ANOVA followed by Tukey’s post hoc test. Data are presented as mean ± SE

We next assessed the effects of intranasal C3a treatment on the therapeutic effects of captopril on preventing epileptogenesis. We found that daily intranasal administration of 1.3 μg/kg C3a partially reversed the therapeutic effects of captopril on the prevention of epileptogenesis, indicated by more frequent seizures and total seizure duration 7 weeks after the KA induction (Seizures frequency: ^&^*p* < 0.05, 2.00 ± 0.44 vs. 0.33 ± 0.20; Total seizure duration: 244.50 ± 79.13 vs. 19.50 ± 12.69, KA + Cap + C3a vs. KA + Cap group, respectively, Fig. 10A, B).

**Fig. 11:**
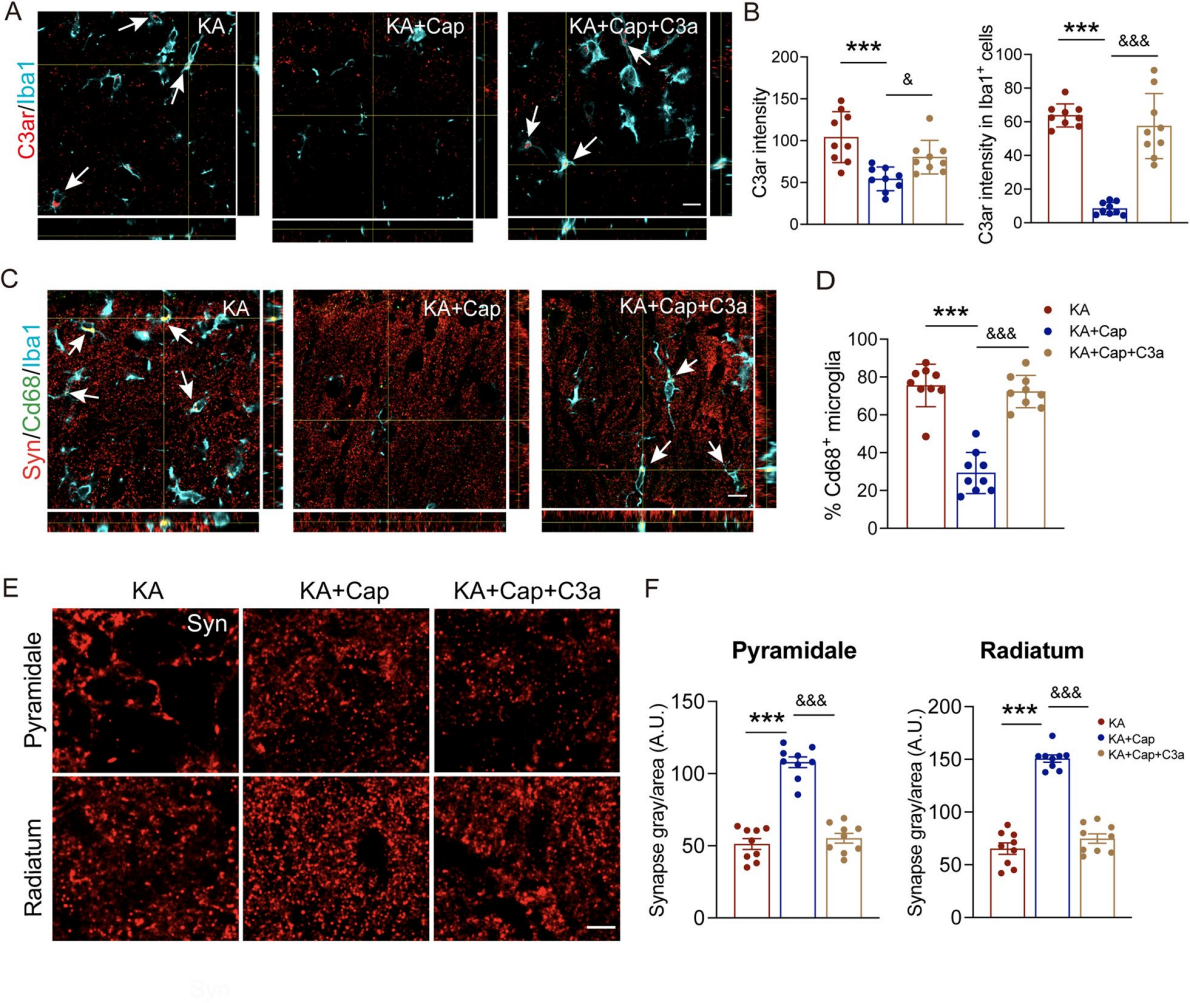
Intranasal instillation of C3a promotes the microglia-mediated synaptic phagocytosis after captopril treatment. **A** Representative anti-C3ar (red) and anti-Iba1 (cyan) double immunostaining indicates that C3a nasal drip reverses the C3ar suppression in microglia following captopril treatment. Scale bar = 10 μm. **B** Quantification of C3ar (red) signal intensity within Iba1^+^ (cyan) microglia is shown. **C**, **D** Immunostaining of Cd68 (green), synapsin (red) and Iba1 (cyan) and quantification of microglia with Cd68^+^ signal in the hippocampal CA1 indicate that C3a significantly reverses the inhibitory effects of captopril on synaptic pruning by microglia. Scale bar = 10 μm. **E** Representative confocal images of synapsin (red) from pyramidale and radiatum layer in the hippocampal CA1 are shown. Scale bar = 5 μm. **F** Quantification of integrated signal intensity synapsin indicates C3a significantly reverses the therapeutic effects of captopril treatment on reducing synaptic loss in the pyramidale and radiatum layer in the hippocampal CA1. *n* = 9 images from 3 rats/group, ***p < 0.001, compared to KA; ^&^*p* < 0.05, ^&&&^*p* < 0.001, compared to KA + Cap, one-way ANOVA followed by Tukey’s post hoc test. Data are presented as mean ± SE

Next, we assessed the effects of intranasal C3a treatment on the therapeutic effects of captopril on glial activation-mediated synaptic remodeling. Co-immunostaining of C3ar and Iba1 revealed a higher level of expression and a higher degree of co-localization in the KA + Cap + C3a group, compared to the KA + Cap group (C3ar intensity: ^&^*p* < 0.05, 80.22 ± 6.56 vs. 54.05 ± 4.72; C3ar within Iba1: ^&&&^*p* < 0.001, 57.45 ± 6.40 vs. 8.40 ± 1.22, KA + Cap + C3a vs. KA + Cap group, Fig. 11A, B). Co-immunostaining of Cd68 with Iba1 revealed that C3a treatment partially blocked the effects of captopril treatment on inhibiting microglia phagocytosis in the hippocampal CA1 (^&&&^*p* < 0.001, 72.33 ± 2.84% vs. 29.26 ± 3.62%, KA + Cap + C3a vs. KA + Cap group, Fig. 11C, D). Moreover, C3a treatment also reversed the therapeutic effects of captopril treatment on synaptic loss in the hippocampal CA1 to a similar level of the KA group (^&&&^*p* < 0.001, pyramidale layer: 55.20 ± 3.37 vs. 107.90 ± 3.72; radiatum layer: 74.82 ± 4.48 vs. 150.70 ± 3.43, KA + Cap + C3a vs. KA + Cap group, Fig. 11E, F). These findings indicate that intranasal C3a treatment starting on the 3^rd^ day following the KA induction contributes to cognitive deficits, epileptogenesis, C3-mediated glial activation and synaptic phagocytosis after the captopril treatment.

## Discussion

In this study, we found that captopril prevented epileptogenesis and improved cognitive impairment in a KA-induced rat model of epilepsy. Captopril treatment inhibited glial activation and interaction, as well as the expression of cytokines and chemokines. Furthermore, captopril treatment reduced complement C3 production in astrocytes while inhibiting C3–C3ar signaling-mediated glial activation, potentially preventing synaptic loss, epileptogenesis, and cognitive impairment. Long-term intranasal administration of recombinant C3a partially reversed the effects of captopril treatment, including the transcriptional signature of epilepsy-related microglia, decreased in markers of synapse, and ultimately reversed the recovery of cognitive function in captopril-treated epileptic rats.

Captopril, an angiotensinase inhibitor, was used in our study to demonstrate the potent effects against epilepsy and improving epilepsy-related cognitive dysfunction, which can be considered a potential drug candidate for treating epilepsy by targeting the ACE. Mechanistically, inhibiting RAS against epilepsy may have anti-convulsant, anti-inflammatory, anti-oxidant, and neuroprotective effects [24]. In the nervous system, dysregulation of classical inflammatory cytokines, such as Il-1β, Tnf-α and Il-6, results in deficits in synaptic plasticity and contributes to neuropathology [43]. In the present study, the mechanism of captopril in the suppression of epilepsy was investigated comprehensively by transcriptomic sequencing technology. In addition to confirming previously reported anti-inflammatory cytokines by qRT-PCR, we discovered inhibitory effects in the rat model on glial cell activation, phagocytosis, and synaptic pruning. Furthermore, the protein interaction networks were used to dissect potential core genes that the captopril treatment regulated. Cd44, one of these genes, has previously been shown to regulate immune and proinflammatory cytokine expression in primary astrocytes and microglia [44]. Mmp9 and Timp1 were found to be overexpressed in neuronal and glial cells and are thought to be biomarkers of blood–brain barrier (BBB) dysfunction in animal models of epilepsy and clinical patients [45, 46]. Captopril treatment normalized the expression of nearly half of the up and down-regulated genes and reversed the microglia and astrocyte phenotypes, according to the sequencing analysis. Furthermore, the results of qPCR and immunofluorescence staining validated the transcriptomic sequencing results. Therefore, our research provides a new drug candidate as well as novel mechanisms of ACEi action in preventing epileptogenesis and improving cognitive function.

One of the key factors contributing to cognitive dysfunction in various neuroinflammatory diseases is glial activation-mediated neuroinflammation and phagocytosis [47]. In the immediate aftermath of seizure induction, astrocytes and microglia were found to interact via the C3–C3ar signaling pathway, which promoted glial activation [15]. Blocking the C3–C3ar signaling pathway decreased glial activation and neuroinflammation, improving seizure outcomes [15]. Using transcriptomic sequencing, we discovered glial activation, neuroinflammation, and synaptic pruning during spontaneous and reflex seizures, which are closer to the genuine context of epileptic seizures. The expression of C3 and C3ar was found to increase in the hippocampus of epileptic rats using qPCR and immunofluorescence staining, accompanied by glial activation, increased contacts between astrocytes and microglia, and elevated Cd68-mediated microglial phagocytosis on synapses. Furthermore, we found a significant reduction in the number of synapses in the hippocampus, which is thought to be highly correlated with epileptogenesis and cognitive impairments. Seizures can cause neuronal or direct synaptic damage, as well as microglia-mediated synaptic pruning, resulting in decreased synapse numbers [48]. The C3 signaling pathway is primarily responsible for the latter [13]. Previous research found that the expression of C3 signaling pathway-related proteins was elevated in clinical epilepsy samples as well as in brain tissues from animal models of epilepsy [16, 49]. Expression of these proteins was negatively correlated with cognitive-behavioral performance in the animal models [49]. We discovered that captopril effectively reversed epilepsy-induced glial activation, neuroinflammation, and synaptic pruning, the order and causality of these effects need to be investigated further. Although the number of synapsin recovered after captopril treatment, the function of synapses needs to be further verified by electrophysiological experiments. Captopril's effects on epilepsy-related glial activation, neuroinflammation, synaptic pruning, epileptogenesis, and cognitive dysfunction were at least partially reversed by administration of the effective C3 cleaved product, C3a. This result suggests that captopril acted at least in part through the C3–C3ar signaling pathway. Furthermore, the mechanism of this pathway is relatively upstream in the various possible mechanisms for the treatment of epilepsy via RAS inhibition. The precise mechanism of ACEi or RAS inhibition on C3–C3ar is unknown. We cannot rule out the possibility that our in vivo findings acted via indirect mechanisms, such as the inhibition of epilepsy-related hypertension [50].

One dose of captopril (50 mg/kg per day, ip) [34] was used in this study to investigate the mechanisms of ACEi on a KA-induced epileptic model in rats. This captopril dose has been widely used in rat pharmacological studies. Captopril's optimal and safe dose should be investigated further in future studies. Captopril has been shown not to enter the brain when administered systemically with the BBB intact [51]. However, the seizures were predisposed to BBB damage [52], which allows captopril to enter the brain via ip administration, implying the feasibility of systemic captopril administration for treating epilepsy in clinical practice. The KA-induced epilepsy model in SD rats is one of the most widely used pharmacological animal models and is relatively reliable for epileptogenesis and cognitive-behavioral assays [53]. We investigated the effects and mechanisms of captopril on epileptogenesis and epilepsy-related cognitive deficits in this study. However, it remains to be seen whether captopril has a therapeutic effect in reversing established epilepsy. Furthermore, C3a activation also influenced gene expression signatures in the brain consistent with activating immune cell activation, as well as increasing neuroinflammation and BBB permeability [54]. Intranasal administration of recombinant human C3a protein has been shown to have brain effects [36], but its specific pharmacokinetics, tissue distribution, and mechanism of action need to be investigated further.

In conclusion, we discovered that captopril inhibited glial activation-related neuroinflammation and synaptic pruning by inhibiting the C3–C3ar signaling pathway, thereby preventing epilepsy formation and cognitive-behavioral impairment. Given the widespread and safe clinical use of captopril, we proposed that it be used in clinics for long-term epilepsy control and cognitive improvement, while recognizing that the efficacy and safety of C3 and C3ar signaling pathway inhibitors remain unknown. In addition, our research uncovered new molecular mechanisms and potential therapeutic targets for preventing epileptogenesis and epilepsy-related cognitive-behavioral disorders.

## Data Availability

The datasets used and/or analyzed during the current study are available from the corresponding author on reasonable request.

## Abbreviations

TLE: Temporal lobe epilepsy
Il-1β: Interleukin-1β
Tnf-α: Tumor necrosis factor-α
RAS: Renin–angiotensin system
CNS: Central nervous system
ACEi: Angiotensin-converting enzyme inhibitor
Ang I: Angiotensin I
AT_1_R: Angiotensin type 1 receptor
LPS: Lipopolysaccharide
SD: Sprague Dawley
KA: Kainic acid
PBS: Phosphate-buffered saline
RNA-seq: RNA sequencing
QC: Quality control
GO: Gene Ontology
KEGG: Kyoto Encyclopedia of Genes and Genomes
GSEA: Gene set enrichment analysis
RT-qPCR: Real-time quantitative polymerase chain reaction
AU: Airy unit
EEG: Electroencephalography
MWM: Morris water maze
NOR: Novel object recognition
PFA: Paraformaldehyde
Gfap: Glial fibrillary acidic protein
Iba1: Ionized calcium-binding adaptor molecule 1
C3: Complement 3
C3ar: Complement 3a receptor
Cd68: Macrophage antigen Cd68
Syn: Synapsin 1/2
SE: Standard error
Mmp9: Matrix metalloproteinase 9
Timp1: TIMP metallopeptidase inhibitor 1
BBB: Blood–brain barrier

## References

[1] Devinsky O, Vezzani A, O'Brien TJ, Jette N, Scheffer IE, de Curtis M, Perucca P (2018). Epilepsy. Nat Rev Dis Primers.

[2] Tellez-Zenteno JF, Hernandez-Ronquillo L (2012). A review of the epidemiology of temporal lobe epilepsy. Epilepsy Res Treat.

[3] Sen A, Capelli V, Husain M (2018). Cognition and dementia in older patients with epilepsy. Brain.

[4] Perucca E, Brodie MJ, Kwan P, Tomson T (2020). 30 years of second-generation antiseizure medications: impact and future perspectives. Lancet Neurol.

[5] Loscher W, Klitgaard H, Twyman RE, Schmidt D (2013). New avenues for anti-epileptic drug discovery and development. Nat Rev Drug Discov.

[6] Devinsky O, Vezzani A, Najjar S, De Lanerolle NC, Rogawski MA (2013). Glia and epilepsy: excitability and inflammation. Trends Neurosci.

[7] Ekdahl CT, Claasen JH, Bonde S, Kokaia Z, Lindvall O (2003). Inflammation is detrimental for neurogenesis in adult brain. Proc Natl Acad Sci USA.

[8] Vitner EB, Farfel-Becker T, Eilam R, Biton I, Futerman AH (2012). Contribution of brain inflammation to neuronal cell death in neuronopathic forms of Gaucher's disease. Brain.

[9] Jiang NM, Cowan M, Moonah SN, Petri WA (2018). The impact of systemic inflammation on neurodevelopment. Trends Mol Med.

[10] Althammer F, Ferreira-Neto HC, Rubaharan M, Roy RK, Patel AA, Murphy A, Cox DN, Stern JE (2020). Three-dimensional morphometric analysis reveals time-dependent structural changes in microglia and astrocytes in the central amygdala and hypothalamic paraventricular nucleus of heart failure rats. J Neuroinflammation.

[11] Boison D, Steinhauser C (2018). Epilepsy and astrocyte energy metabolism. Glia.

[12] Liddelow SA, Guttenplan KA, Clarke LE, Bennett FC, Bohlen CJ, Schirmer L, Bennett ML, Munch AE, Chung WS, Peterson TC, Wilton DK, Frouin A, Napier BA, Panicker N, Kumar M, Buckwalter MS, Rowitch DH, Dawson VL, Dawson TM, Stevens B, Barres BA (2017). Neurotoxic reactive astrocytes are induced by activated microglia. Nature.

[13] Andoh M, Ikegaya Y, Koyama R (2019). Synaptic pruning by microglia in epilepsy. J Clin Med.

[14] Bialas AR, Stevens B (2013). TGF-beta signaling regulates neuronal C1q expression and developmental synaptic refinement. Nat Neurosci.

[15] Wei Y, Chen T, Bosco DB, Xie M, Zheng J, Dheer A, Ying Y, Wu Q, Lennon VA, Wu LJ (2021). The complement C3–C3aR pathway mediates microglia-astrocyte interaction following status epilepticus. Glia.

[16] Wyatt SK, Witt T, Barbaro NM, Cohen-Gadol AA, Brewster AL (2017). Enhanced classical complement pathway activation and altered phagocytosis signaling molecules in human epilepsy. Exp Neurol.

[17] Litvinchuk A, Wan YW, Swartzlander DB, Chen F, Cole A, Propson NE, Wang Q, Zhang B, Liu Z, Zheng H (2018). Complement C3aR inactivation attenuates tau pathology and reverses an immune network deregulated in tauopathy models and Alzheimer's disease. Neuron.

[18] Young KG, Yan K, Picketts DJ (2019). C3aR signaling and gliosis in response to neurodevelopmental damage in the cerebellum. J Neuroinflammation.

[19] Wang C, Yue H, Hu Z, Shen Y, Ma J, Li J, Wang XD, Wang L, Sun B, Shi P, Wang L, Gu Y (2020). Microglia mediate forgetting via complement-dependent synaptic elimination. Science.

[20] Arganaraz GA, Konno AC, Perosa SR, Santiago JFC, Boim MA, Vidotti DB, Varella PPV, Costa LG, Canzian M, Porcionatto MA, Yacubian EM, Sakamoto AC, Carrete H, Centeno RS, Amado D, Cavalheiro EA, Silva JA, Mazzacoratti MDGN (2008). The renin-angiotensin system is upregulated in the cortex and hippocampus of patients with temporal lobe epilepsy related to mesial temporal sclerosis. Epilepsia.

[21] Benicky J, Sanchez-Lemus E, Pavel J, Saavedra JM (2009). Anti-inflammatory effects of angiotensin receptor blockers in the brain and the periphery. Cell Mol Neurobiol.

[22] Sun H, Wu H, Yu X, Zhang G, Zhang R, Zhan S, Wang H, Bu N, Ma X, Li Y (2015). Angiotensin II and its receptor in activated microglia enhanced neuronal loss and cognitive impairment following pilocarpine-induced status epilepticus. Mol Cell Neurosci.

[23] Hammer A, Stegbauer J, Linker RA (2017). Macrophages in neuroinflammation: role of the renin-angiotensin-system. Pflugers Arch.

[24] Ivanova N, Tchekalarova J (2019). The potential therapeutic capacity of inhibiting the brain renin-angiotensin system in the treatment of co-morbid conditions in epilepsy. CNS Drugs.

[25] Asraf K, Torika N, Apte RN, Fleisher-Berkovich S (2018). Microglial activation is modulated by captopril: in vitro and in vivo studies. Front Cell Neurosci.

[26] Tchekalarova JD, Ivanova N, Atanasova D, Pechlivanova DM, Lazarov N, Kortenska L, Mitreva R, Lozanov V, Stoynev A (2016). Long-term treatment with losartan attenuates seizure activity and neuronal damage without affecting behavioral changes in a model of co-morbid hypertension and epilepsy. Cell Mol Neurobiol.

[27] Ivanova NM, Atanasova D, Pechlivanova DM, Mitreva R, Lazarov N, Stoynev AG, Tchekalarova JD (2015). Long-term intracerebroventricular infusion of angiotensin II after kainate-induced status epilepticus: Effects on epileptogenesis, brain damage, and diurnal behavioral changes. Epilepsy Behav.

[28] Senatorov VV, Friedman AR, Milikovsky DZ, Ofer J, Saar-Ashkenazy R, Charbash A, Jahan N, Chin G, Mihaly E, Lin JM, Ramsay HJ, Moghbel A, Preininger MK, Eddings CR, Harrison HV, Patel R, Shen Y, Ghanim H, Sheng H, Veksler R, Sudmant PH, Becker A, Hart B, Rogawski MA, Dillin A, Friedman A, Kaufer D (2019). Blood-brain barrier dysfunction in aging induces hyperactivation of TGFbeta signaling and chronic yet reversible neural dysfunction. Sci Transl Med..

[29] Bar-Klein G, Cacheaux LP, Kamintsky L, Prager O, Weissberg I, Schoknecht K, Cheng P, Kim SY, Wood L, Heinemann U, Kaufer D, Friedman A (2014). Losartan prevents acquired epilepsy via TGF-beta signaling suppression. Ann Neurol.

[30] Semis M, Gugiu GB, Bernstein EA, Bernstein KE, Kalkum M (2019). The Plethora of angiotensin-converting enzyme-processed peptides in Mouse plasma. Anal Chem.

[31] AbdAlla S, El Hakim A, Abdelbaset A, Elfaramawy Y, Quitterer U (2015). Inhibition of ACE retards tau hyperphosphorylation and signs of neuronal degeneration in aged rats subjected to chronic mild stress. Biomed Res Int.

[32] Maroso M, Balosso S, Ravizza T, Liu J, Aronica E, Iyer AM, Rossetti C, Molteni M, Casalgrandi M, Manfredi AA, Bianchi ME, Vezzani A (2010). Toll-like receptor 4 and high-mobility group box-1 are involved in ictogenesis and can be targeted to reduce seizures. Nat Med.

[33] Nestor J, Arinuma Y, Huerta TS, Kowal C, Nasiri E, Kello N, Fujieda Y, Bialas A, Hammond T, Sriram U, Stevens B, Huerta PT, Volpe BT, Diamond B (2018). Lupus antibodies induce behavioral changes mediated by microglia and blocked by ACE inhibitors. J Exp Med.

[34] Boskabadi J, Askari VR, Hosseini M, Boskabady MH (2019). Immunomodulatory properties of captopril, an ACE inhibitor, on LPS-induced lung inflammation and fibrosis as well as oxidative stress. Inflammopharmacology.

[35] Ondetti MA, Rubin B, Cushman DW (1977). Design of specific inhibitors of angiotensin-converting enzyme: new class of orally active antihypertensive agents. Science.

[36] Stokowska A, Atkins AL, Moran J, Pekny T, Bulmer L, Pascoe MC, Barnum SR, Wetsel RA, Nilsson JA, Dragunow M, Pekna M (2017). Complement peptide C3a stimulates neural plasticity after experimental brain ischaemia. Brain.

[37] Love MI, Huber W, Anders S (2014). Moderated estimation of fold change and dispersion for RNA-seq data with DESeq2. Genome Biol.

[38] Chen T, Bosco DB, Ying Y, Tian DS, Wu LJ (2021). The emerging role of microglia in neuromyelitis optica. Front Immunol.

[39] Young K, Morrison H (2018). Quantifying microglia morphology from photomicrographs of immunohistochemistry prepared tissue using ImageJ. J Vis Exp.

[40] Dunn KW, Kamocka MM, McDonald JH (2011). A practical guide to evaluating colocalization in biological microscopy. Am J Physiol Cell Physiol.

[41] Schidlitzki A, Bascunana P, Srivastava PK, Welzel L, Twele F, Tollner K, Kaufer C, Gericke B, Feleke R, Meier M, Polyak A, Ross TL, Gerhauser I, Bankstahl JP, Johnson MR, Bankstahl M, Loscher W (2020). Proof-of-concept that network pharmacology is effective to modify development of acquired temporal lobe epilepsy. Neurobiol Dis.

[42] Presumey J, Bialas AR, Carroll MC (2017). Complement system in neural synapse elimination in development and disease. Adv Immunol.

[43] Vezzani A, Lang B, Aronica E (2015). Immunity and Inflammation in Epilepsy. Cold Spring Harb Perspect Med.

[44] Wang Y, Li L, Wu Y, Zhang S, Ju Q, Yang Y, Jin Y, Shi H, Sun C (2022). CD44 deficiency represses neuroinflammation and rescues dopaminergic neurons in a mouse model of Parkinson's disease. Pharmacol Res.

[45] Bronisz E, Kurkowska-Jastrzebska I (2016). Matrix metalloproteinase 9 in epilepsy: the Role of neuroinflammation in seizure development. Mediators Inflamm.

[46] Ruber T, David B, Luchters G, Nass RD, Friedman A, Surges R, Stocker T, Weber B, Deichmann R, Schlaug G, Hattingen E, Elger CE (2018). Evidence for peri-ictal blood-brain barrier dysfunction in patients with epilepsy. Brain.

[47] Kwon HS, Koh SH (2020). Neuroinflammation in neurodegenerative disorders: the roles of microglia and astrocytes. Transl Neurodegener.

[48] Alexander A, Maroso M, Soltesz I (2016). Organization and control of epileptic circuits in temporal lobe epilepsy. Prog Brain Res.

[49] Schartz ND, Wyatt-Johnson SK, Price LR, Colin SA, Brewster AL (2018). Status epilepticus triggers long-lasting activation of complement C1q–C3 signaling in the hippocampus that correlates with seizure frequency in experimental epilepsy. Neurobiol Dis.

[50] Lukawski K, Jakubus T, Raszewski G, Czuczwar SJ (2010). Captopril potentiates the anticonvulsant activity of carbamazepine and lamotrigine in the mouse maximal electroshock seizure model. J Neural Transm (Vienna).

[51] Migdalof BH, Antonaccio MJ, McKinstry DN, Singhvi SM, Lan SJ, Egli P, Kripalani KJ (1984). Captopril: pharmacology, metabolism and disposition. Drug Metab Rev.

[52] Ndode-Ekane XE, Hayward N, Grohn O, Pitkanen A (2010). Vascular changes in epilepsy: functional consequences and association with network plasticity in pilocarpine-induced experimental epilepsy. Neuroscience.

[53] Loscher W (2017). Animal models of seizures and epilepsy: past, present, and future role for the discovery of antiseizure drugs. Neurochem Res.

[54] Propson NE, Roy ER, Litvinchuk A, Kohl J, Zheng H (2021). Endothelial C3a receptor mediates vascular inflammation and blood-brain barrier permeability during aging. J Clin Invest.

